# A comprehensive analysis of all-cause and cause-specific excess deaths in 30 countries during 2020

**DOI:** 10.1007/s10654-023-01044-x

**Published:** 2023-09-08

**Authors:** Gianfranco Alicandro, Carlo La Vecchia, Nazrul Islam, Margherita Pizzato

**Affiliations:** 1 Department of Pathophysiology and Transplantation, Università degli Studi di Milano, Milan, Italy; 2 Cystic Fibrosis Centre, Fondazione IRCCS Ca’ Granda Ospedale Maggiore Policlinico, Milan, Italy; 3 Department of Clinical Sciences and Community Health, Università degli Studi di Milano, Milan, Italy; 4 Nuffield Department of Population Health, University of Oxford, Oxford, UK; 5 School of Primary Care, Population Sciences and Medical Education, University of Southampton, Southampton, UK

**Keywords:** SARS-CoV-2, COVID-19, Mortality, Excess deaths, Cardiovascular diseases, Diabetes

## Abstract

The impact of COVID-19 on mortality from specific causes of death remains poorly understood. This study analysed cause-of-death data provided by the World Health Organization from 2011 to 2019 to estimate excess deaths in 2020 in 30 countries. Over-dispersed Poisson regression models were used to estimate the number of deaths that would have been expected if the pandemic had not occurred, separately for men and women. The models included year and age categories to account for temporal trends and changes in size and age structure of the populations. Excess deaths were calculated by subtracting observed deaths from expected ones. Our analysis revealed significant excess deaths from ischemic heart diseases (IHD) (in 10 countries), cerebrovascular diseases (CVD) (in 10 countries), and diabetes (in 19 countries). The majority of countries experienced excess mortality greater than 10%, including Mexico (+ 38·8% for IHD, + 34·9% for diabetes), Guatemala (+ 30·0% for IHD, + 10·2% for CVD, + 39·7% for diabetes), Cuba (+ 18·8% for diabetes), Brazil (+ 12·9% for diabetes), the USA (+ 15·1% for diabetes), Slovenia (+ 33·8% for diabetes), Poland (+ 30·2% for IHD, + 19·5% for CVD, + 26 1% for diabetes), Estonia (+ 26·9% for CVD, + 34·7% for diabetes), Bulgaria (+ 22·8% for IHD, + 11·4% for diabetes), Spain (+ 19·7% for diabetes), Italy (+ 18·0% for diabetes), Lithuania (+ 17·6% for diabetes), Finland (+ 13·2% for diabetes) and Georgia (+ 10·7% for IHD, + 19·0% for diabetes). In 2020, 22 out of 30 countries had a significant increase in total mortality. Some of this excess was attributed to COVID-19, but a substantial increase was also observed in deaths attributed to cardiovascular diseases and diabetes.

**Supplementary Information:**

The online version contains supplementary material available at 10.1007/s10654-023-01044-x.

## Introduction

During the first two years of the pandemic 5.4 million deaths were reported worldwide due to Coronavirus Disease (COVID-19) [1]. This figure involves uncertainty and does not cover the full burden of the pandemic, as it leaves out the number of deaths that were not officially attributed to COVID-19 and it also ignores the indirect effects resulting from the disruption and reorganization of the healthcare systems during the pandemic [2].

It soon became clear that the number of COVID-19 deaths was not a good indicator of the real impact of the pandemic. Thus, researchers turned their attention to total excess deaths, defined as the difference between the number of observed deaths from any cause and the number of deaths that would have been expected on the basis of past trends [2]. This indicator is not affected by underdiagnosis and underreporting, and it also measures the indirect effects of the pandemic on other health conditions. However, total excess, being an indicator of overall impact, cannot provide any clue on the non-COVID conditions that have been the most affected by the pandemic.

Since the start of the pandemic, international organizations and independent research groups have made several attempts to estimate total excess deaths [1, 3–5]. However, information on excess deaths from non-COVID-19 causes, as well as on disparities by sex and age group, remains sparse due to the limited availability of data.

It is also important to consider the impact of COVID-19 in different countries, as the excess in total mortality has varied widely, ranging from none in Australia and New Zealand to > 50% in some Latin American countries [1].

In this study, we provide estimates of excess mortality from some major non-COVID-19 causes in 2020 for 30 countries.

## Data sources and methods

We used publicly available data on death counts by cause and population data by sex, age and country for the period 2011–2020. Mortality and population data were downloaded from the World Health Organization (WHO) mortality database [6]. When population data for the whole period were not available in the WHO database, we used the EUROSTAT database for the European countries and the United Nations database for the rest of the countries [7, 8].

We considered total mortality and mortality from selected major groups of causes of death, aggregated according to the International Statistical Classification of Diseases and Related Health Problems, 10^th^ Revision (ICD-10): neoplasms (ICD-10 codes: C00-D48), diabetes (ICD-10 codes: E10-E14), influenza and pneumonia (ICD-10: J09-J18), dementia and Alzheimer disease (ICD-10 codes: F00-F03, G30), all circulatory system diseases (ICD-10: I00-I99), ischemic heart diseases (ICD-10 codes: I20-I25), cerebrovascular diseases (ICD-10 codes: I60-I69), transport accidents (ICD-10 codes: V01-V99, Y85), suicides (ICD-10 codes: X60-X84, Y87.0) and ill-defined causes (ICD-10 codes: R00-R94, R96-R99). These groups of causes were chosen based on previous reports that showed possible increased risks or worsening of management during the pandemic periods [9–14]. The ICD-10 was also used to retrieve the number of deaths attributed to COVID-19 (ICD-10 codes: U07.1, U07.2, U09.9, U10.9).

We selected countries with complete (annually reported) mortality and population data from 2011 to 2020. To mitigate the influence of random fluctuations in mortality data caused by the small number of events for some of the causes considered, we excluded countries with < 10,000 deaths reported in 2020. Additionally, we excluded countries with completeness of cause-of-death data below 90% [15]. Completeness indicates the percentage of the total number of estimated deaths in the country that has been registered with cause-of-death information in the vital registration system.

We calculated the number of excess deaths in each country and for each group of causes by comparing the observed deaths in 2020 with the number of deaths that would have been expected if the pandemic had not occurred. To estimate the expected number of deaths we used the following over-dispersed Poisson regression model:

$${\text{log}}\left( {E\left( {Deaths_{i,j} } \right)} \right) = \beta_{0} + \beta_{1} Year_{i} + \beta_{2} Age_{j} + {\text{log}}\left( {Pop_{i,j} } \right)$$

The model was trained on a period spanning from 2011 to 2019, separately for each sex, country and cause of death. The model included: a linear term for calendar years to capture temporal trends in mortality; age groups to account for the effect of age on mortality risk; and the natural logarithm of the population as offset term. The offset term was used to account for the size of the population and changes in its age structure over the study period.

To obtain combined estimates for all the 30 countries considered, we extended the model by adding a main effect for country and a year-by-country interaction term:

$$\begin{aligned} \log \left( {E\left( {Deaths_{i,j,k} } \right)} \right) & = \beta_{0} + \beta_{1} Year_{i} + \beta_{2} Age_{j} + \beta_{3} Country_{k} \\ & \quad + \beta_{4} (Country_{k} \times Year_{i} ) + {\text{log}}\left( {Pop_{i,j,k} } \right) \\ \end{aligned}$$

Estimates obtained using the described model were then aggregated to have the expected deaths in all countries.

Due to the small number of events at young ages for some causes of death, we used three different classifications for age: 1) 0–4, 5–9, …, 85 + for all causes; 2) 0–39, 40–44, …, 85 + for selected causes of deaths excluding dementia and Alzheimer disease and 3) 0–59, 60–64, …, 85 + for dementia and Alzheimer disease. The coefficients of the models were derived by fitting the models on 2011–2019 data, i.e. before the outbreak of the pandemic, and then used to compute the number of expected deaths in 2020.

Excess deaths were presented both in absolute terms (i.e. the difference between observed and expected deaths) and relative terms (i.e. the percent relative differences, also known as P-score), and with empirical 95% confidence intervals (CI) obtained through a Monte Carlo simulation. We sampled 10,000 iterations from a multivariate normal distribution for each set of the model’s coefficients using the parameter estimates and the variance–covariance matrix. For each iteration, we computed the difference between the observed and the expected deaths and obtained the 95% CI using the normal approximation. Excess death estimates were considered statistically significant when the 95% CI of the point estimates did not include zero.

Due to the varying mortality risks associated with COVID-19 and other analysed conditions across different sex and age categories [16], we conducted a stratified analysis by sex and age groups (< 75 vs ≥ 75 years) in countries with populations of at least 10 million in 2020. We presented these results separately for countries with high-income and upper-middle-income economies, according to the World Bank classification [17].

To assess the robustness of our estimates to natural fluctuations in mortality from year to year, we performed a sensitivity analysis. We calculated the difference between the number of observed deaths and the expected deaths in 2019, using historical data from the preceding period (2011–2018). The expected deaths were estimated using the same model employed in the main analysis. We obtained P-scores based on these estimates, which were then compared with the P-scores obtained in 2020.

The analysis was conducted using the statistical software R (R version 4.2.0).

## Results

Among the 140 countries in the WHO mortality database, 48 had complete mortality data from 2011 to 2020. After excluding countries with incomplete population data, those with less than 10,000 deaths in 2020 and those with completeness of cause-of-death data below 90%, 30 countries were included in our analysis. The flowchart outlining the procedure for the country selection is provided in the Supplementary Information.

The study analysed 18 European countries (Austria, Bulgaria, the Czech Republic, Denmark, Estonia, Finland, Georgia, Germany, Italy, Latvia, Lithuania, the Netherlands, Poland, Serbia, Slovenia, Spain, Switzerland and the UK), 3 countries from South America (Argentina, Brazil, Chile), 4 countries from Central and North America (Mexico, Cuba, Guatemala, and the USA), 4 Asian and Oceanian countries (Israel, Japan, the Republic of Korea and Australia), and one African country (Mauritius).

Table 1 shows the total number of deaths registered in 2020, those attributed to COVID-19, and the estimates of the excess deaths in each of the countries included in the study. In Bulgaria, Italy, the Netherlands, Poland, Serbia, Slovenia, Spain, Switzerland, the UK, Argentina, Brazil, Mexico and the USA the mortality rates from COVID-19 were ≥ 10 per 10,000 population, while rates < 1 per 10,000 were reported in Mauritius, Cuba, Japan, the Republic of Korea and Australia. For all selected countries combined, we estimated approximately 1.4 million excees deaths from any cause in 2020, corresponding to an excess mortality in relative terms of + 12.2%. Mexico had the highest excess in all-cause mortality in 2020 (+ 43·1%). The USA, Chile, and some European countries, including Bulgaria, the Czech Republic, Italy, Poland, Serbia, Slovenia and Spain, had an excess of around + 15%, while an excess of around 10% was observed in Guatemala, Lithuania, the Netherlands, Switzerland, and the UK. Austria, Estonia, Finland, Israel, Estonia, Latvia and Argentina showed lower excesses, whereas no significant excess was detected in Denmark, Georgia, Germany, Mauritius, Cuba, Japan, the Republic of Korea, and Australia. These excesses were not observed in 2019 (Supplementary Information).

**Table 1:** Absolute and percent differences in the number of deaths from any cause registered in 2020 relative to the expected deaths, COVID-19 deaths and 2020 population, by country

Country	Observed deaths	Expected deaths	Absolute difference (95% CI)	Percent difference (95% CI)	COVID-19 deaths	COVID-19 deaths/absolute difference	COVID-19 mortality rate (per 10,000)	Population (thousands)
Austria	91,599	85,135	6464 (5197; 7730)	7.6 (6.1; 9.1)	6491	1.00	7.3	8916.8
Bulgaria	124,735	108,018	16,717 (15,476; 17,957)	15.5 (14.3; 16.6)	8554	0.51	12.3	6934.0
Czech Republic	129,289	112,025	17,264 (15,946; 18,581)	15.4 (14.2; 16.6)	10,539	0.61	9.8	10,700.2
Denmark	54,399	54,560	− 161 (− 1042; 720)	− 0.3 (− 1.9; 1.3)	1070	− 6.65	1.8	5824.7
Estonia	15,679	15,246	433 (124; 741)	2.8 (0.8; 4.9)	203	0.47	1.5	1329.0
Finland	55,498	54,673	825 (175; 1474)	1.5 (0.3; 2.7)	558	0.68	1.0	5525.3
Georgia	50,537	50,959	− 422 (− 2799; 1955)	− 0.8 (− 5.5; 3.8)	2587	− 6.13	6.9	3722.7
Germany	985,572	975,408	10,164 (− 2125; 22,453)	1.0 (− 0.2; 2.3)	39,758	3.91	4.8	83,160.9
Italy	740,443	641,248	99,195 (92,531; 105,858)	15.5 (14.4; 16.5)	78,141	0.79	13.1	59,641.5
Latvia	28,589	27,557	1032 (582; 1481)	3.7 (2.1; 5.4)	700	0.68	3.7	1900.4
Lithuania	43,547	38,904	4643 (3932; 5353)	11.9 (10.1; 13.8)	2266	0.49	8.1	2794.9
Netherlands	168,678	153,118	15,560 (13,489; 17,630)	10.2 (8.8; 11.5)	20,173	1.30	11.5	17,512.1
Poland	477,355	408,539	68,816 (64,736; 72,895)	16.8 (15.8; 17.8)	41,451	0.60	10.9	37,929.7
Serbia	116,774	100,300	16,474 (15,419; 17,528)	16.4 (15.4; 17.5)	10,352	0.63	15.0	6899.1
Slovenia	24,016	20,718	3298 (2911; 3684)	15.9 (14.1; 17.8)	3390	1.03	16.1	2100.1
Spain	493,776	424,718	69,058 (64,177; 73,938)	16.3 (15.1; 17.4)	74,839	1.08	15.8	47,355.7
Switzerland	76,195	68,550	7645 (6931; 8358)	11.2 (10.1; 12.2)	9294	1.22	10.8	8606.0
UK	688,805	627,838	60,967 (54,076; 67,857)	9.7 (8.6; 10.8)	81,354	1.33	12.1	67,078.5
Mauritius	11,060	11,166	− 106 (− 351; 139)	− 0.9 (− 3.1; 1.2)	10	− 0.09	0.1	1265.7
Argentina	370,413	344,271	26,142 (21,977; 30,306)	7.6 (6.4; 8.8)	52,344	2.00	11.6	45,195.8
Brazil	1,554,141	1,375,064	179,077 (167,981; 190,172)	13.0 (12.2; 13.8)	212,671	1.19	10.0	212,559.4
Chile	126,150	109,942	16,208 (14,990; 17,425)	14.7 (13.6; 15.8)	18,680	1.15	9.8	19,116.2
Cuba	112,384	113,138	− 754 (− 2135; 627)	− 0.7 (− 1.9; 0.6)	143	− 0.19	0.1	11,326.6
Guatemala	95,660	87,348	8312 (6879; 9744)	9.5 (7.9; 11.2)	7980	0.96	4.5	17,915.6
Mexico	1,065,607	744,746	320,861 (311,287; 330,434)	43.1 (41.8; 44.4)	200,200	0.62	15.5	128,932.8
USA	3,383,613	2,923,919	459,694 (433,207; 486,180)	15.7 (14.8; 16.6)	350,827	0.76	10.6	331,002.6
Israel	48,797	46,474	2323 (1621; 3024)	5.0 (3.5; 6.5)	3160	1.36	3.4	9215.1
Japan	1,372,317	1,368,778	3539 (− 9330; 16,408)	0.3 (− 0.7; 1.2)	3466	0.98	0.3	123,399.0
Republic of Korea	304,921	302,146	2775 (− 1076; 6626)	0.9 (− 0.4; 2.2)	950	0.34	0.2	51,349.3
Australia	161,293	169,284	− 7991 (− 9692; − 6289)	− 4.7 (− 5.7; − 3.7)	899	− 0.11	0.4	25,655.3
All countries	12,971,842	11,561,798	1,410,044 (1,256,997; 1,563,090)	12.2 (10.9; 13.5)	1,243,050	0.88	9.2	1,354,865

In absolute terms, the USA and Mexico had the highest number of excess deaths, with approximately 460 and 321 thousand additional deaths, respectively. Among the European countries, Italy was the country with the highest number of excess deaths (~ 99.000 deaths) followed by Spain (~ 69.000 deaths) and the UK (~ 61.000 deaths).

Out of the 22 countries which had excess mortality in 2020, 12 had a ratio of COVID-19 deaths to estimated excess deaths lower than one, indicating that reported COVID-19 did not account for all the excess deaths. Conversely, in countries with ratios above one, reported COVID-19 deaths exceeded the estimated total excess deaths. Some countries showed negative values, indicating that the observed number of deaths was below the expected number.

An overall excess mortality from ischemic heart diseases of approximately 86,000 deaths (+ 7.3%) was estimated for all the 30 countries considered (Table 2). A significant excess was detected in 10 countries, including Mexico (+ 38·8%), Poland (+ 30·2%), Guatemala (+ 30·0%), Bulgaria (+ 22·8%), Georgia (+ 10·7%), Cuba (+ 8·5%), the USA (+ 6·8%), the Czech Republic (+ 6·1%), Italy (+ 5·2%) and Lithuania (+ 4·3%). Poland and Bulgaria showed higher mortality from ischemic heart diseases also in 2019 compared to what was expected (+ 13.6% in Poland and + 7.5% in Bulgaria). However, the excess mortality in 2019 was considerably lower than that observed in 2020 (Supplementary Information).

**Table 2:** Absolute and percent differences in the number of deaths from ischemic heart diseases registered in 2020 relative to the expected deaths, by country

Country	Observed deaths	Expected deaths	Absolute difference (95% CI)	Percent difference (95% CI)
Austria	13,445	13,442	3 (− 418; 424)	0 (− 3.1; 3.2)
Bulgaria	15,244	12,417	2827 (2293; 3360)	22.8 (18.5; 27.1)
Czech Republic	23,353	22,002	1351 (701; 2000)	6.1 (3.2; 9.1)
Denmark	3328	3194	134 (− 18; 286)	4.2 (− 0.6; 9.0)
Estonia	2119	2400	− 281 (− 394; − 167)	− 11.7 (− 16.4; − 7.0)
Finland	8600	8764	− 164 (− 427; 99)	− 1.9 (− 4.9; 1.1)
Georgia	5787	5227	560 (110; 1009)	10.7 (2.1; 19.3)
Germany	121,462	122,219	− 757 (− 4177; 2663)	− 0.6 (− 3.4; 2.2)
Italy	63,356	60,216	3140 (1593; 4686)	5.2 (2.6; 7.8)
Latvia	6586	6350	236 (− 16; 488)	3.7 (− 0.3; 7.7)
Lithuania	14,216	13,625	591 (84; 1097)	4.3 (0.6; 8.1)
Netherlands	8037	7811	226 (− 105; 557)	2.9 (− 1.3; 7.1)
Poland	54,403	41,774	12,629 (10,783; 14,474)	30.2 (25.8; 34.6)
Serbia	8791	8782	9 (− 375; 393)	0.1 (− 4.3; 4.5)
Slovenia	1768	1856	− 88 (− 195; 19)	− 4.7 (− 10.5; 1.0)
Spain	29,654	29,220	434 (− 619; 1487)	1.5 (− 2.1; 5.1)
Switzerland	6822	6623	199 (− 19; 417)	3.0 (− 0.3; 6.3)
UK	64,050	61,879	2171 (− 428; 4770)	3.5 (− 0.7; 7.7)
Mauritius	1313	1320	− 7 (− 123; 109)	− 0.5 (− 9.3; 8.3)
Argentina	22,520	22,808	− 288 (− 1549; 973)	− 1.3 (− 6.8; 4.3)
Brazil	109,490	120,286	− 10,796 (− 19,415; − 2176)	− 9 (− 16.1; − 1.8)
Chile	7959	8341	− 382 (− 881; 117)	− 4.6 (− 10.6; 1.4)
Cuba	18,781	17,305	1476 (785; 2166)	8.5 (4.5; 12.5)
Guatemala	8050	6194	1856 (1192; 2519)	30.0 (19.2; 40.7)
Mexico	163,685	117,889	45,796 (37,640; 53,951)	38.8 (31.9; 45.8)
USA	382,803	358,268	24,535 (7421; 41,648)	6.8 (2.1; 11.6)
Israel	3701	3514	187 (− 1; 375)	5.3 (0; 10.7)
Japan	67,278	65,314	1964 (− 255; 4183)	3.0 (− 0.4; 6.4)
Republic of Korea	14,055	14,321	− 266 (− 1033; 501)	− 1.9 (− 7.2; 3.5)
Australia	16,587	17,698	− 1111 (− 1962; − 259)	− 6.3 (− 11.1; − 1.5)
All countries	1,267,243	1,180,755	86,488 (63,596; 109,379)	7.3 (5.4; 9.3)

The overall estimate indicate a 2.1% excess mortality from cerebrovascular diseases when considering all countries combined. However, 10 countries showed higher excesses, including Estonia (+ 26·9%), Poland (+ 19·5%), Guatemala (+ 10·2%), Bulgaria (+ 8·7%), Cuba (+ 6·3%), Czech Republic (+ 4·7%), Italy (+ 4·7%), Serbia (+ 4·6%), the USA (+ 4·1%) and Spain (+ 3·3%) (Table 3). Estonia showed higher mortality than expected from cerebrovascular diseases also in 2019 (+ 39.9%) (Supplementary Information). Estimates of all cardiovascular causes are given in the Supplementary Information.

**Table 3:** Absolute and percent differences in the number of deaths from cerebrovascular diseases registered in 2020 relative to the expected deaths, by country

Country	Observed deaths	Expected deaths	Absolute difference (95% CI)	Percent difference (95% CI)
Austria	4737	4582	155 (− 26; 336)	3.4 (− 0.6; 7.3)
Bulgaria	22,010	20,254	1756 (1141; 2370)	8.7 (5.6; 11.7)
Czech Republic	7552	7213	339 (99; 578)	4.7 (1.4; 8.0)
Denmark	3007	3133	− 126 (− 261; 9)	− 4.0 (− 8.3; 0.3)
Estonia	1093	861	232 (164; 299)	26.9 (19.0; 34.7)
Finland	4026	3953	73 (− 93; 239)	1.8 (− 2.4; 6.0)
Georgia	10,146	10,079	67 (− 652; 786)	0.7 (− 6.5; 7.8)
Germany	53,308	53,833	− 525 (− 1894; 844)	− 1.0 (− 3.5; 1.6)
Italy	57,061	54,493	2568 (1394; 3741)	4.7 (2.6; 6.9)
Latvia	5120	5369	− 249 (− 476; − 21)	− 4.6 (− 8.9; − 0.4)
Lithuania	5226	5049	177 (− 40; 394)	3.5 (− 0.8; 7.8)
Netherlands	8850	9371	− 521 (− 850; − 191)	− 5.6 (− 9.1; − 2.0)
Poland	32,540	27,221	5319 (4214; 6423)	19.5 (15.5; 23.6)
Serbia	9851	9420	431 (121; 740)	4.6 (1.3; 7.9)
Slovenia	1939	1955	− 16 (− 111; 79)	− 0.8 (− 5.7; 4.0)
Spain	25,817	25,004	813 (157; 1468)	3.3 (0.6; 5.9)
Switzerland	3524	3396	128 (− 3; 259)	3.8 (− 0.1; 7.6)
UK	34,460	34,478	− 18 (− 1162; 1126)	− 0.1 (− 3.4; 3.3)
Mauritius	948	1001	− 53 (− 154; 48)	− 5.3 (− 15.4; 4.8)
Argentina	17,479	19,985	− 2506 (− 3628; − 1383)	− 12.5 (− 18.2; − 6.9)
Brazil	98,812	100,774	− 1962 (− 8094; 4170)	− 1.9 (− 8.0; 4.1)
Chile	7948	7806	142 (− 265; 549)	1.8 (− 3.4; 7.0)
Cuba	10,975	10,325	650 (249; 1050)	6.3 (2.4; 10.2)
Guatemala	3606	3273	333 (32; 633)	10.2 (1.0; 19.3)
Mexico	36,182	35,513	669 (− 1358; 2696)	1.9 (− 3.8; 7.6)
USA	160,262	153,993	6269 (522; 12,015)	4.1 (0.3; 7.8)
Israel	2178	2103	75 (− 45; 195)	3.6 (− 2.1; 9.3)
Japan	102,960	101,521	1439 (− 1764; 4642)	1.4 (− 1.7; 4.6)
Republic of Korea	21,858	21,491	367 (− 670; 1404)	1.7 (− 3.1; 6.5)
Australia	9469	9807	− 338 (− 704; 28)	− 3.4 (− 7.2; 0.3)
All countries	762,944	746,960	15,984 (7407; 24,560)	2.1 (1.0; 3.3)

Approximately 76,000 excess deaths were estimated for diabetes in the 30 countries included in the analysis, accounting for an excess mortality of + 18.3% (Table 4). Significant excesses were detected in 19 countries, including Guatemala (+ 39·7%), Mexico (+ 34·9%), Estonia (+ 34·7%), Slovenia (+ 33·8%), Poland (+ 26·1%), Spain (+ 19·7%), Georgia (+ 19·0%), Cuba (+ 18·8%), Italy (+ 18·0%), Lithuania (+ 17·6%), the USA (+ 15·1%), Finland (+ 13·2%), Brazil (+ 12·9%), Bulgaria (+ 11·4%), the UK (+ 8·6%), Israel (+ 6·7%), Serbia (+ 6·1%), Germany (+ 4·6%), and Japan (+ 3·7%). Slovenia, Lithuania and Georgia also experienced higher mortality from diabetes than expected in 2019, with excesses of + 24 2%, + 22 9% and + 28 6%, respectively (Supplementary Information).

**Table 4:** Absolute and percent differences in the number of deaths from diabetes registered in 2020 relative to the expected deaths, by country

Country	Observed deaths	Expected deaths	Absolute difference (95% CI)	Percent difference (95% CI)
Austria	2855	3077	− 222 (− 382; − 61)	− 7.2 (− 12.4; − 2.0)
Bulgaria	1769	1588	181 (74; 287)	11.4 (4.7; 18.1)
Czech Republic	4993	4780	213 (− 58; 484)	4.5 (− 1.2; 10.1)
Denmark	1253	1368	− 115 (− 209; − 20)	− 8.4 (− 15.3; − 1.5)
Estonia	334	248	86 (46; 125)	34.7 (18.5; 50.4)
Finland	667	589	78 (19; 136)	13.2 (3.2; 23.1)
Georgia	977	821	156 (63; 248)	19.0 (7.7; 30.2)
Germany	25,807	24,673	1134 (420; 1847)	4.6 (1.7; 7.5)
Italy	25,513	21,627	3886 (3274; 4497)	18.0 (15.1; 20.8)
Latvia	700	638	62 (0; 123)	9.7 (0.0; 19.3)
Lithuania	688	585	103 (29; 176)	17.6 (5.0; 30.1)
Netherlands	2799	2690	109 (− 31; 249)	4.1 (− 1.2; 9.3)
Poland	12,160	9640	2520 (2062; 2977)	26.1 (21.4; 30.9)
Serbia	3394	3200	194 (14; 373)	6.1 (0.4; 11.7)
Slovenia	443	331	112 (74; 149)	33.8 (22.4; 45.0)
Spain	11,297	9440	1857 (1563; 2150)	19.7 (16.6; 22.8)
Switzerland	1011	1197	− 186 (− 263; − 108)	− 15.5 (− 22.0; − 9.0)
UK	8902	8200	702 (267; 1136)	8.6 (3.3; 13.9)
Mauritius	2310	2321	− 11 (− 189; 167)	− 0.5 (− 8.1; 7.2)
Argentina	9800	9582	218 (− 329; 765)	2.3 (− 3.4; 8.0)
Brazil	75,704	67,081	8623 (4880; 12,365)	12.9 (7.3; 18.4)
Chile	3665	3928	− 263 (− 604; 78)	− 6.7 (− 15.4; 2.0)
Cuba	2887	2430	457 (307; 606)	18.8 (12.6; 24.9)
Guatemala	9996	7154	2842 (1961; 3722)	39.7 (27.4; 52.0)
Mexico	148,596	110,129	38,467 (29,948; 46,985)	34.9 (27.2; 42.7)
USA	102,187	88,785	13,402 (7869; 18,934)	15.1 (8.9; 21.3)
Israel	2598	2435	163 (30; 295)	6.7 (1.2; 12.1)
Japan	13,899	13,401	498 (6; 989)	3.7 (0; 7.4)
Republic of Korea	8455	8167	288 (− 135; 711)	3.5 (− 1.7; 8.7)
Australia	5148	5098	50 (− 202; 302)	1.0 (− 4.0; 5.9)
All countries	490,807	414,864	75,943 (65,184; 86,701)	18.3 (15.7; 20.9)

An excess mortality was observed from influenza and pneumonia in Bulgaria (+ 120·2%), Mexico (+ 83·5%), and Serbia (+ 57·0%); from dementia and Alzheimer’s disease in Chile (+ 16·5%), Poland (+ 16·2%), Slovenia (+ 16·2%) and the USA (+ 6·1%). Deaths assigned to ill-defined causes also increased in 20 countries, including Mauritius (+ 302%), Latvia (+ 95·2%), the Czech Republic (+ 84·5%), Italy (+ 64·5%), Australia (+ 69·0%), Serbia (+ 57·4%), the USA (+ 39·9%), Brazil (+ 26·8%), Mexico (+ 24·5%), the Republic of Korea (+ 21·9%), the Netherlands (+ 22·8%), the UK (+ 17·7%), Argentina (+ 16·9%), Denmark (+ 14%), Bulgaria (+ 12·3%), Switzerland (+ 8·5%) and Austria (+ 8·4%) (Supplementary Information).

**Fig. 1:**
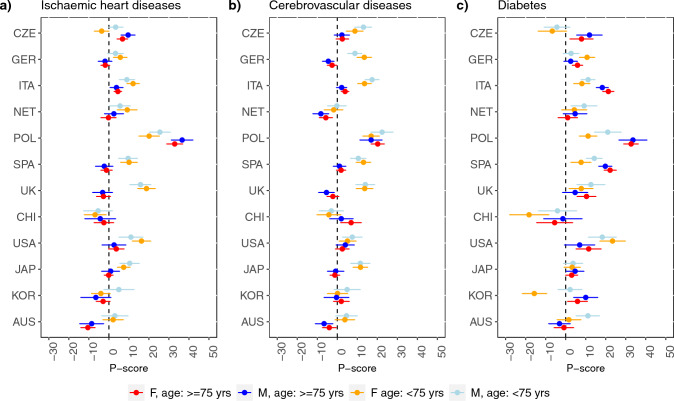
Percent difference between observed and expected deaths (P-score) from ischemic heart diseases, cerebrovascular disease and diabetes by sex and age group (< 75 vs. ≥ 75 years), in 12 high-income countries (gross national income per capita of $13,205 or more) with population size ≥ 10 million. CZE: Czech Republic. GER: Germany. ITA: Italy. NET: Netherlands. POL: Poland. SPA: Spain. UK: United Kingdom. CHI: Chile. USA: United States of America. JAP: Japan. KOR: Republic of Korea. AUS: Australia

**Fig. 2:**
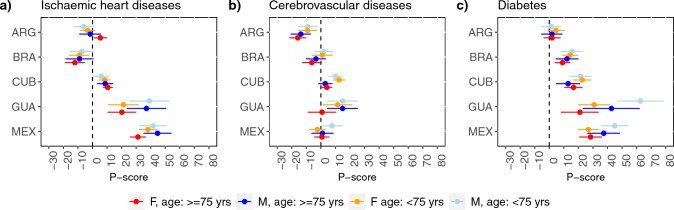
Percent difference between observed and expected deaths (P-score) from ischemic heart diseases, cerebrovascular diseases and diabetes by sex and age group (< 75 vs. ≥ 75 years), in 5 upper-middle-income countries (gross national income per capita between $4256 and $13,205) with population size ≥ 10 million. ARG: Argentina. BRA: Brazil. CUB: Cuba. GUA: Guatemala. MEX: Mexico

Figure 1 shows the percent difference between observed and expected deaths (P-score) from ischemic heart diseases, cerebrovascular diseases and diabetes in 2020 by sex and age group (< 75 vs. ≥ 75 years) in the 12 high-income countries with a population size ≥ 10 million. Corresponding data for upper-middle-income countries are shown in Fig. 2.

There were no clear differences by sex, except for diabetes in Guatemala and Mexico (only for ages < 75 years) where males had higher excess mortality. In Spain, the UK, the USA and Japan, a significant excess mortality from ischemic heart diseases was observed only at ages < 75 years, as was the case for cerebrovascular diseases in the Czech Republic, Germany, Italy, Spain, the UK, and Japan. Excess mortality from diabetes was higher at ages ≥ 75 years in the Czech Republic, Italy, Poland, Spain, and the Republic of Korea compared to younger ages. Detailed estimates for all the causes considered are reported in the Supplementary Information.

## Discussion

This study assessed mortality from non-COVID-19 conditions in 30 countries during the first year of the pandemic. It revealed significant increases in mortality from ischemic heart and cerebrovascular diseases in about a third of the selected countries, as well as from diabetes in the majority of the countries considered. These increases were generally more pronounced in South American countries and, for diabetes, also in Eastern European ones. It also found that in high-income countries, the excess mortality from cardiovascular diseases affected individuals aged < 75 years to a greater extent. In most countries, there were excesses in ill-defined causes of death, while no excess was observed in cancer mortality.

Substantial variation in cause-specific excess mortality emerged between countries with advanced and well-organized healthcare systems, such as Denmark, Germany, Japan, and Australia, and other high-income countries like the USA and Italy, whose healthcare systems were inadequately prepared and severely affected by the pandemic.

Consistent with previous studies, our research has revealed that a significant proportion of excess deaths in 2020 were not attributed to COVID-19 [18, 19]. For example, in the USA, a study evaluating excess mortality from March 1, 2020 to January 2, 2021, found that only 72% of the overall excess mortality has been attributed to COVID-19. Our results, which indicate that 76% of excess deaths were attributed to COVID-19, are consistent with that estimate. This underestimation, although to varying degrees, has been observed in other countries as well, raising doubts about whether the excess deaths from causes other than COVID-19 can be partly attributed to errors in the certification of the cause of death.

Data on cause-specific excess deaths are limited to a few countries where different methods have been used to estimate expected deaths [9–14]. Most studies rely on the average number of deaths registered in a period preceding the pandemic, thus ignoring temporal trends and changes in size and structure of the population. Despite the differences in methodologies, these studies reported a consistent excess in diabetes-related deaths in Italy (+ 19%) [10], the USA (+ 16%) [14], the Republic of Korea (+ 10·4%) [13] and Mexico (+ 36·8%) [12]. Excesses of 5% for heart and 7% for cerebrovascular diseases were estimated in the USA, [14] and similar excesses for all cardiovascular diseases were reported in Poland (+ 5·9%) [20]. Higher excesses were found in Mexico for ischemic heart (+ 32·5%) and hypertensive diseases (+ 25·0%) [12].

During the initial phase of the pandemic, it became apparent that patients with pre-existing cardiovascular diseases and related risk factors, such as hypertension and diabetes were at greater risk of developing severe COVID-19. Although younger patients with COVID-19 have a lower prevalence of cardiovascular risk factors, those who have hypertension, diabetes, or pre-existing cardiovascular diseases were also at relatively higher risk of poor outcomes compared to older patients [21]. The main reasons for this remain to be clarified, but it has been suggested that young patients may have experienced a higher occurrence of inflammation-related complications and may have been less aware of cardiovascular symptoms [22]. Other major risk factors, including smoking, dyslipidemia and arterial hypertension are associated to a greater relative risk of cardiovascular events in the middle-aged than in the elderly [23].

SARS-CoV-2 infection can also lead to the development of de novo cardiovascular conditions, and a history of COVID-19 should be considered a cardiovascular risk factor per se.

COVID-19 has been linked to cardiovascular dysfunction and complications, which can occur both in the acute and post-acute phases of the infection [24]. Up to 62% of COVID-19 hospitalized patients experience acute cardiac injury, as indicated by elevated cardiac biomarkers. This condition, when associated with thromboembolic disorders, can lead to fatal outcomes during the early phase of the infection [25]. Beyond the first 30 days and up to one year, patients remained at higher risk of developing a range of cardiovascular conditions, including myocardial infarction, heart failure, arrhythmias, myocarditis, pericarditis, stroke and thromboembolic disorders [26]. This increased risk may contribute to the persistently higher all-cause mortality observed in patients after the acute phase of the infection. According to a study based on UK Biobank data, COVID-19 patients are approximately 10 times more likely to die from any cause after 30 days from infection and up to an average follow-up of 141 days as compared to uninfected controls [27]. Long-term cardiac sequelae of COVID-19 have also been observed through cardiac magnetic resonance imaging, showing evidence of myocardial fibrosis, and functional impairment [28].

The underlying mechanisms of cardiovascular dysfunction and heart injury are complex and multifactorial, including direct viral injury of cardiac cells and indirect pathological mechanisms such as hypoxia-induced myocardial injury, and mechanisms mediated by the immune response such as vasculitis, endotheliitis, microvascular injury and thrombosis [29].

The excess mortality from diabetes suggests the coexistence of two pathological pathways: (1) COVID-19 leads to de novo diabetes in individuals who may not have developed it otherwise; (2) COVID-19 can accelerate the progression to diabetes in patients with pre-existing metabolic disorders. The diabetogenic effect of COVID-19 has been related to inflammation and oxidative stress, which can lead to pleiotropic alterations in glucose metabolism, including impaired insulin activity and insulin resistance [30]. Additionally, high-dose corticosteroid therapy used to manage lung damage caused by SARS-CoV-2 infection may have contributed to rapid deterioration of glycaemic control, resulting in a new-onset diabetes or exacerbating pre-existing metabolic disorders [31].

As the pandemic has unfolded, health services and social care sectors have faced mounting pressure, leading to a contraction of healthcare delivery for chronic diseases. With medical resources mostly redirected to assist COVID-19 patients, non-COVID-19 ones have experienced difficulties in accessing healthcare services. Fear of contracting COVID-19 and a sense of vulnerability have also discouraged individuals from seeking healthcare services, especially during lockdown periods. The pandemic presented significant challenges for patients requiring careful daily management, such as those with diabetes. Without professional guidance, some may have struggled to control their glycaemic levels, adhere to treatment, and manage complications of the disease. For other health conditions, such as cancer, the lower screening rates, and delays in diagnosis and treatment during the pandemic may have had a negative impact on prognosis, with effects that may only be seen in the decades to come [32].

The varying impact observed in each country can be attributed to several factors, including: (1) the timing of implementing, intensity and effectiveness of non-pharmaceutical measures to contain the transmission, such as restricting internal movements and gatherings, the closing of schools, workplaces and shops and mandating mask-wearing [33]; (2) the ability to promptly track COVID-19 cases and implement epidemiological monitoring; (3) the different age structures and prevalence of comorbidities in each country; (4) the different resources available for managing and treating COVID-19 and its complications; (5) the diagnostic capacity for recognizing new cardiovascular events after infection.

When interpreting our results, it is important to note that certification of causes of death is prone to underreporting. The extent to which underreporting impacted our estimate of excess mortality is difficult to quantify, but it is likely that diabetes was particularly affected. This is because diabetes is often considered a comorbidity rather than a direct cause of death with less than 50% of the death certificates of diabetic patients reporting diabetes as cause of death [34]. During the early phases of the pandemic, proper certification of deaths became even more difficult, likely resulting in the assignment of some deaths to ill-defined causes, for example to ICD-10 codes within the chapter of symptoms, signs and abnormal clinical and laboratory findings, as well as to unspecified pneumonia. This can be inferred from the high number of excess deaths attributed to these causes in certain countries, which is unlikely to be ascribed to random fluctuations. For example, deaths from pneumonia and influenza in Bulgaria were 3025 in 2020, compared to a range of 1296 to 1497 during the previous period (2011–2019). Similarly, in Mauritius, there were 514 deaths from ill-defined causes in 2020, compared to a range of 127 to 214 deaths reported in previous years (2011–2019). In the Czech Republic, there were 3676 deaths from ill-defined causes in 2020, whereas the range in the previous years was between 1137 and 1908 deaths. Moreover, in some countries, there were fewer deaths than expected for certain groups of causes, and this could be attributed to COVID-19 having a competing effect over other causes of death. As a result, our estimates probably underestimate the true impact of the pandemic on cardiovascular mortality and mortality from diabetes.

To test the robusteness of our estimates, we compared the cause-specific expected deaths with the observed deaths in 2019. No significant differences were overall observed, except for some excesses in some Eastern European countries, which, however were lower than those estimated for 2020. Improvement in the certification of causes of death could partly account for the observed excess in 2019.

Our estimates differ from those provided by other studies. Various attempts have been made to estimate excess mortality during the pandemic period, all based on the difference between observed deaths and expected deaths. The main difference lies in how the expected deaths were calculated. Most estimates use statistical models that incorporate multiple factors known to affect mortality, such as sex, age, population size, temporal trend, seasonality, heatwaves, and influenza outbreaks. These models may consider some of these factors. Another crucial factor affecting the observed differences is the choice of the baseline period used to train the model for expected deaths [35]. In our study, we used yearly mortality data covering a period of 9 years, from 2011 to 2019. Other studies used different models and baseline periods, resulting in similar results in some countries while varying in others. For instance, in a study we published in 2021 based on weekly mortality data, using a baseline period spanning from 2016 to 2019 and excluding weeks affected by heatwaves and influenza outbreaks, the total excess deaths for Germany, Italy and Spain were estimated at 25,900, 89,100 and 84,100, respectively [5]. That study was conducted using data collected during the first months of 2021 when countries only provided provisional data that were subsequently updated in later releases. According to the WHO, the estimates of total excess deaths in 2020, obtained using annual mortality data and a baseline period of 2015–2019, were 55,648 in Germany, 100,431 in Italy, and 72,576 in Spain. While our current estimate significantly differs from the WHO's estimate for Germany, the estimates for Italy and Spain align closely with those reported in our study.

The significant variation in estimates for Germany has been previously noted by two German researchers who, using actuarial science methods, estimated approximately 4000 excess deaths for 2020 [36]. Furthermore, our estimate of excess mortality for Germany (+ 1% in relative terms) aligns with the estimate provided in the work by Levitt et al., where all possible combinations of baseline periods were averaged [35].

Determining which approach outperforms the others is challenging. Nevertheless, a longer baseline period allows for a more comprehensive assessment of the trend in mortality rate and helps mitigate the effects of perturbations caused by heatwaves and influenza outbreaks, without completely eliminating them.

Finally, we measured the impact of the pandemic on causes of deaths other than COVID-19 during a period when vaccines were not available, treatment options were limited, the circulating SARS-CoV-2 variants were different from those observed in subsequent years, and healthcare systems were overwhelmed. Today, the situation has greatly improved, and the number of severe cases of COVID-19 has dramatically reduced. However, it remains to be determined whether vaccinated patients who were infected by the variants circulating in 2021 and 2022 are still at an increased risk of mortality from cardiovascular diseases and diabetes.

## Conclusions

During the first year of the pandemic, when vaccines were not available, there was a significant excess mortality in 22 out of the 30 countries considered in this study. A significant increase was also observed in deaths related to cardiovascular diseases and diabetes. The specific contributions of indirect pandemic effects versus direct effects of SARS-CoV-2 infection remain uncertain and require further investigation.

## Author contributors

GA, MP and CLV conceived and designed the study. GA and MP directly accessed and verified the underlying data in this study. GA performed the data analysis. GA and MP drafted the original manuscript. CLV and NI contributed with critical input and revision to the manuscript. CLV was responsible for funding acquisition. All authors had full access to all the data in the study and accept responsibility to submit for publication.

### Supplementary Information

Below is the link to the electronic supplementary material.

Supplementary file1 (PDF 1150 KB)

## Data Availability

All data used in this study are publicly available and have been cited in detail in the appendix.
