# Supplementary material for: A comprehensive analysis of all-cause and cause-specific excess deaths in 30 countries during 2020
Source: Eur J Epidemiol. 2023 Sep 8;38(11):1153–64. doi: 10.1007/s10654-023-01044-x (PMC10663248; doi:10.1007/s10654-023-01044-x)

Supplementary Information

Title: A comprehensive analysis of all-cause and cause-specific excess deaths in 30 countries during 2020

Figure 1. Flowchart of the country selection. 2

Countries abbreviations: 3

Table 1. Absolute and percent differences in the number of deaths from neoplasms registered in 2020 relative to the expected deaths, by country. 4

Table 2. Absolute and percent differences in the number of deaths from cardiovascular diseases registered in 2020 relative to the expected deaths, by country. 5

Table 3. Absolute and percent differences in the number of deaths from influenza and pneumonia registered in 2020 relative to the expected deaths, by country. 6

Table 4. Absolute and percent differences in the number of deaths from dementia and Alzheimer disease registered in 2020 relative to the expected deaths, by country. 7

Table 5. Absolute and percent differences in the number of deaths from transport accidents registered in 2020 relative to the expected deaths, by country. 8

Table 6. Absolute and percent differences in the number of deaths from suicides registered in 2020 relative to the expected deaths, by country. 9

Table 7. Absolute and percent differences in the number of deaths from ill-defined causes registered in 2020 relative to the expected deaths, by country. 10

Table 8. Absolute and percent differences in the number of deaths from any cause registered in 2020 relative to the expected deaths and COVID-19 deaths, by sex and age group (<75 vs ≥75 years) in countries with populations of ≥10 million. 11

Table 9. Absolute and percent differences in the number of deaths from neoplasms registered in 2020 relative to the expected deaths, by age group (<75 vs ≥75 years) in countries with population of ≥10 million. 13

Table 10. Absolute and percent differences in the number of deaths from cardiovascular diseases registered in 2020 relative to the expected deaths, by age group (<75 vs ≥75 years) in countries with population ≥10 million. 15

Table 11. Absolute and percent differences in the number of deaths from ischemic heart diseases registered in 2020 relative to the expected deaths, by age group (<75 vs ≥75 years) in countries with population of ≥10 million. 17

Table 12. Absolute and percent differences in the number of deaths from cerebrovascular diseases registered in 2020 relative to the expected deaths, by age group (<75 vs ≥75 years) in countries with population ≥10 million. 19

Table 13. Absolute and percent differences in the number of deaths from diabetes registered in 2020 relative to the expected deaths, by age group (<75 vs ≥75 years) in countries with population ≥10 million. 21

Table 14. Absolute and percent differences in the number of deaths from influenza and pneumonia registered in 2020 relative to the expected deaths, by age group (<75 vs ≥75 years) in countries with population ≥10 million. 23

Table 15. Absolute and percent differences in the number of deaths from dementia and Alzheimer disease registered in 2020 relative to the expected deaths, by age group (<75 vs ≥75 years) in countries with population ≥10 million. 25

Table 16. Absolute and percent differences in the number of deaths from transport accidents registered in 2020 relative to the expected deaths, by age group (<75 vs ≥75 years) in countries with population ≥10 million. 27

Table 17. Absolute and percent differences in the number of deaths from suicides registered in 2020 relative to the expected deaths, by age group (<75 vs ≥75 years) in countries with population ≥10 million. 29

Table 18. Absolute and percent differences in the number of deaths from ill-defined causes registered in 2020 relative to the expected deaths, by age group (<75 vs ≥75 years) in countries with population ≥10 million. 31

Figure 2. Sensitivity analysis: comparison of the relative difference between observed and expected deaths (P-score) in 2019 vs 2020. 33

Figure 1. Flowchart of the country selection.

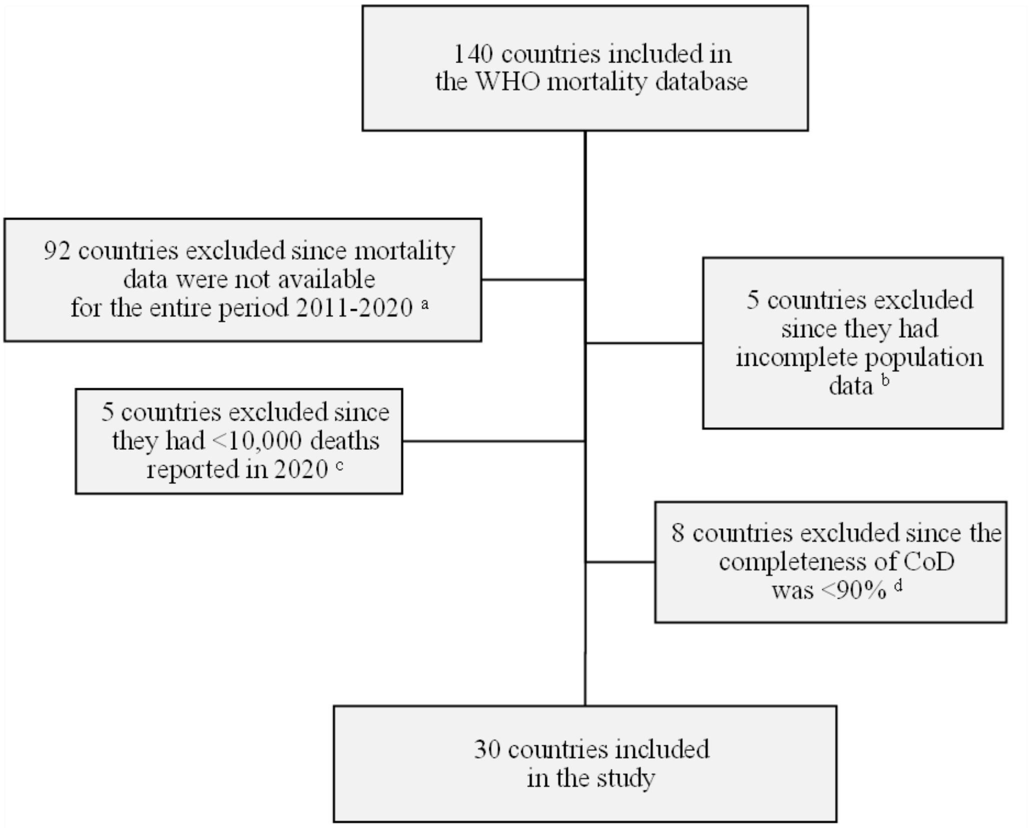

CoD: Causes of death

[a] Andorra, Anguilla, Antigua and Barbuda, Azerbaijan, Bahamas, Bahrain, Barbados, Belarus, Belgium, Belize, Bermuda, Bolivia, Bosnia and Herzegovina, British Virgin Islands, Brunei Darussalam, Canada, Cape Verde, Cayman Islands, Croatia, Dominican Republic, Egypt, El Salvador, Fiji, France, French Guiana, Greece, Guadeloupe, Guyana, Haiti, Honduras, Hong Kong SAR, Hungary, Iran (Islamic Republic of), Iraq, Ireland, Jamaica, Jordan, Kiribati, Kuwait, Kyrgyzstan, Lebanon, Libyan Arab Jamahiriya, Maldives, Malta, Martinique, Mayotte, Mongolia, Montenegro, Morocco, Netherlands Antilles, New Zealand, Norway, Occupied Palestinian Territory, Oman, Panama, Philippines, Portugal, Puerto Rico, Qatar, Réunion, Republic of Moldova, Rodrigues, Romania, Russian Federation, Saint Kitts and Nevis, Saint Pierre and Miquelon, San Marino, Saudi Arabia, Serbia and Montenegro Former, Seychelles, Singapore, Slovakia, Solomon Islands, South Africa, Sri Lanka, Suriname, Sweden, Syrian Arab Republic, Tajikistan, TFYR Macedonia, Thailand, Trinidad and Tobago, Tunisia, Turkey, Turkmenistan, Turks and Caicos Islands, Ukraine, United Arab Emirates, Uruguay, Uzbekistan, Venezuela and Virgin Islands (USA).

[b] Armenia, Aruba, Dominica Montserrat and Paraguay

[c] Grenada, Iceland, Luxembourg, Saint Lucia and Saint Vincent and Grenadines

[d] Colombia, Costa Rica, Cyprus, Ecuador, Kazakhstan, Malaysia, Nicaragua and Peru

Countries abbreviations:

ARG: Argentina
AUS: Australia
AU: Austria
BRA: Brazil
BUL: Bulgaria
CHI: Chile
CUB: Cuba
CZE: Czech Republic
DEN: Denmark
EST: Estonia
FIN: Finland
GEO: Georgia
GER: Germany
GUA: Guatemala
ISR: Israel
ITA: Italy
JAP: Japan
KOR: Republic of Korea
LAT: Latvia
LIT: Lithuania
MAU: Mauritius
MEX: Mexico
NET: Netherlands
POL: Poland
SER: Serbia
SLO: Slovenia
SPA: Spain
SWI: Switzerland
UK: United Kingdom
USA: United States of America

Table 1. Absolute and percent differences in the number of deaths from neoplasms registered in 2020 relative to the expected deaths, by country.

Country	Observed deaths	Expected deaths	Absolute difference (95% CI)	Percent difference (95% CI)
AU	21,803	21,337	466 (-279; 1211)	2.2 (-1.3; 5.7)
BUL	18,527	17,743	784 (-95; 1663)	4.4 (-0.5; 9.4)
CZE	28,716	28,324	392 (-644; 1428)	1.4 (-2.3; 5.0)
DEN	16,153	16,277	-124 (-734; 486)	-0.8 (-4.5; 3.0)
EST	3,801	4,025	-224 (-408; -39)	-5.6 (-10.1; -1.0)
FIN	13,411	13,343	68 (-412; 548)	0.5 (-3.1; 4.1)
GEO	8,089	9,054	-965 (-1537; -392)	-10.7 (-17.0; -4.3)
GER	239,552	242,408	-2856 (-10199; 4487)	-1.2 (-4.2; 1.9)
ITA	176,438	178,811	-2373 (-7561; 2815)	-1.3 (-4.2; 1.6)
LAT	6,123	6,086	37 (-225; 299)	0.6 (-3.7; 4.9)
LIT	8,424	8,289	135 (-280; 550)	1.6 (-3.4; 6.6)
NET	47,089	47,240	-151 (-1836; 1534)	-0.3 (-3.9; 3.2)
POL	108,702	111,118	-2416 (-6636; 1804)	-2.2 (-6.0; 1.6)
SER	21,386	22,015	-629 (-1578; 320)	-2.9 (-7.2; 1.5)
SLO	6,436	6,541	-105 (-399; 189)	-1.6 (-6.1; 2.9)
SPA	112,741	113,048	-307 (-3916; 3302)	-0.3 (-3.5; 2.9)
SWI	17,506	18,145	-639 (-1241; -36)	-3.5 (-6.8; -0.2)
UK	172,301	174,425	-2124 (-8634; 4386)	-1.2 (-4.9; 2.5)
MAU	1,429	1,524	-95 (-218; 28)	-6.2 (-14.3; 1.8)
ARG	61,062	65,929	-4867 (-9108; -625)	-7.4 (-13.8; -0.9)
BRA	229,286	242,055	-12,769 (-27,764; 2226)	-5.3 (-11.5; 0.9)
CHI	28,656	28,878	-222 (-1545; 1101)	-0.8 (-5.4; 3.8)
CUB	27,194	27,046	148 (-909; 1205)	0.5 (-3.4; 4.5)
GUA	8,944	8,570	374 (-513; 1261)	4.4 (-6.0; 14.7)
MEX	95,461	96,450	-989 (-7620; 5642)	-1 (-7.9; 5.8)
USA	618,620	621,831	-3211 (-29,899; 23,477)	-0.5 (-4.8; 3.8)
ISR	12,182	12,321	-139 (-857; 579)	-1.1 (-7.0; 4.7)
JAP	391,521	388,176	3345 (-7488; 14178)	0.9 (-1.9; 3.7)
KOR	83,771	83,538	233 (-3566; 4032)	0.3 (-4.3; 4.8)
AUS	48,788	49,980	-1192 (-3249; 865)	-2.4 (-6.5; 1.7)
All countries	*2,634,112*	*2,664,469*	*-30,357 (-125,583; 64,869)*	*-1.1 (-4.7; 2.4)*

Table 2. Absolute and percent differences in the number of deaths from cardiovascular diseases registered in 2020 relative to the expected deaths, by country.

Country	Observed deaths	Expected deaths	Absolute difference (95% CI)	Percent difference (95% CI)
AU	32,544	32,750	-206 (-1108; 696)	-0.6 (-3.4; 2.1)
BUL	74,648	69,257	5391 (3352; 7429)	7.8 (4.8; 10.7)
CZE	50,344	46,158	4186 (2920; 5451)	9.1 (6.3; 11.8)
DEN	11,415	11,860	-445 (-854; -35)	-3.8 (-7.2; -0.3)
EST	7,701	7,596	105 (-150; 360)	1.4 (-2.0; 4.7)
FIN	18,566	18,277	289 (-267; 845)	1.6 (-1.5; 4.6)
GEO	21,895	23,357	-1462 (-3020; 96)	-6.3 (-12.9; 0.4)
GER	338,001	342,533	-4532 (-13,001; 3937)	-1.3 (-3.8; 1.1)
ITA	225,244	220,807	4437 (-503; 9377)	2 (-0.2; 4.2)
LAT	15,408	15,183	225 (-429; 879)	1.5 (-2.8; 5.8)
LIT	22,940	21,284	1656 (824; 2487)	7.8 (3.9; 11.7)
NET	35,281	36,370	-1089 (-2162; -15)	-3 (-5.9; 0.0)
POL	174,399	160,489	13,910 (8437; 19,382)	8.7 (5.3; 12.1)
SER	51,040	49,395	1645 (310; 2979)	3.3 (0.6; 6.0)
SLO	7,714	8,010	-296 (-566; -25)	-3.7 (-7.1; -0.3)
SPA	119,853	117,166	2687 (-320; 5694)	2.3 (-0.3; 4.9)
SWI	20,511	20,279	232 (-270; 734)	1.1 (-1.3; 3.6)
UK	151,641	147,757	3884 (-1299; 9067)	2.6 (-0.9; 6.1)
MAU	3,302	3,811	-509 (-819; -198)	-13.4 (-21.5; -5.2)
ARG	95,664	96,152	-488 (-4726; 3750)	-0.5 (-4.9; 3.9)
BRA	357,550	368,596	-11,046 (-33,903; 11,811)	-3 (-9.2; 3.2)
CHI	29,018	28,523	495 (-783; 1773)	1.7 (-2.7; 6.2)
CUB	44,376	41,639	2737 (1233; 4240)	6.6 (3.0; 10.2)
GUA	16,452	13,812	2640 (1535; 3744)	19.1 (11.1; 27.1)
MEX	254,791	198,449	56,342 (44,549; 68,134)	28.4 (22.4; 34.3)
USA	912,653	881,524	31,129 (-9008; 71,266)	3.5 (-1.0; 8.1)
ISR	10,339	10,058	281 (-92; 654)	2.8 (-0.9; 6.5)
JAP	344,780	342,439	2341 (-6587; 11,269)	0.7 (-1.9; 3.3)
KOR	60,042	62,317	-2275 (-4833; 283)	-3.7 (-7.8; 0.5)
AUS	40,288	42,891	-2603 (-4249; -956)	-6.1 (-9.9; -2.2)
All countries	*3,548,400*	*3,437,607*	*110,793 (40,253; 181,332)*	*3.2 (1.2; 5.3)*

Table 3. Absolute and percent differences in the number of deaths from influenza and pneumonia registered in 2020 relative to the expected deaths, by country.

Country	Observed deaths	Expected deaths	Absolute difference (95% CI)	Percent difference (95% CI)
AU	1,201	1,690	-489 (-636; -341)	-28.9 (-37.6; -20.2)
BUL	3,025	1,374	1651 (1488; 1813)	120.2 (108.3; 132.0)
CZE	3,931	3,909	22 (-167; 211)	0.6 (-4.3; 5.4)
DEN	1,543	1,926	-383 (-497; -268)	-19.9 (-25.8; -13.9)
EST	271	392	-121 (-174; -67)	-30.9 (-44.4; -17.1)
FIN	117	349	-232 (-284; -179)	-66.5 (-81.4; -51.3)
GEO	2,726	2,463	263 (-35; 561)	10.7 (-1.4; 22.8)
GER	17,206	21,836	-4630 (-5411; -3848)	-21.2 (-24.8; -17.6)
ITA	15,837	16,201	-364 (-912; 184)	-2.2 (-5.6; 1.1)
LAT	378	487	-109 (-176; -41)	-22.4 (-36.1; -8.4)
LIT	612	697	-85 (-172; 2)	-12.2 (-24.7; 0.3)
NET	3,021	3,593	-572 (-839; -304)	-15.9 (-23.4; -8.5)
POL	19,913	19,545	368 (-437; 1173)	1.9 (-2.2; 6.0)
SER	3,034	1,933	1101 (961; 1240)	57 (49.7; 64.1)
SLO	301	472	-171 (-220; -121)	-36.2 (-46.6; -25.6)
SPA	9,662	11,948	-2286 (-2756; -1815)	-19.1 (-23.1; -15.2)
SWI	1,217	1,741	-524 (-634; -413)	-30.1 (-36.4; -23.7)
UK	21,879	31,169	-9290 (-10,440; -8139)	-29.8 (-33.5; -26.1)
MAU	190	306	-116 (-160; -71)	-37.9 (-52.3; -23.2)
ARG	26,968	37,596	-10,628 (-12,557; -8698)	-28.3 (-33.4; -23.1)
BRA	68,194	90,616	-22,422 (-30,094; -14,749)	-24.7 (-33.2; -16.3)
CHI	3,209	4,708	-1499 (-1893; -1104)	-31.8 (-40.2; -23.4)
CUB	6,646	9,082	-2436 (-2934; -1937)	-26.8 (-32.3; -21.3)
GUA	4,068	6,643	-2575 (-5109; -40)	-38.8 (-76.9; -0.6)
MEX	57,277	31,208	26,069 (21330; 30,807)	83.5 (68.3; 98.7)
USA	53,542	54,325	-783 (-3464; 1898)	-1.4 (-6.4; 3.5)
ISR	1,485	1,628	-143 (-253; -32)	-8.8 (-15.5; -2.0)
JAP	79,401	94,668	-15,267 (-17,695; -12,838)	-16.1 (-18.7; -13.6)
KOR	22,509	28,681	-6172 (-6961; -5382)	-21.5 (-24.3; -18.8)
AUS	2,287	4,249	-1962 (-2211; -1712)	-46.2 (-52.0; -40.3)
All countries	*431,650*	*485,287*	*-53,637 (-88,681; -18,592)*	*-11.1 (-18.3; -3.8)*

Table 4. Absolute and percent differences in the number of deaths from dementia and Alzheimer disease registered in 2020 relative to the expected deaths, by country.

Country	Observed deaths	Expected deaths	Absolute difference (95% CI)	Percent difference (95% CI)
AU	3,665	4,829	-1164 (-1442; -885)	-24.1 (-29.9; -18.3)
BUL	146	121	25 (0; 50)	20.7 (0.0; 41.3)
CZE	4,353	4,281	72 (-237; 381)	1.7 (-5.5; 8.9)
DEN	4,205	4,903	-698 (-968; -427)	-14.2 (-19.7; -8.7)
EST	227	197	30 (-8; 68)	15.2 (-4.1; 34.5)
FIN	10,667	11,099	-432 (-886; 22)	-3.9 (-8.0; 0.2)
GEO	139	138	1 (-37; 39)	0.7 (-26.8; 28.3)
GER	60,693	70,557	-9864 (-12,445; -7282)	-14 (-17.6; -10.3)
ITA	37,395	38,020	-625 (-2011; 761)	-1.6 (-5.3; 2.0)
LAT	577	670	-93 (-171; -14)	-13.9 (-25.5; -2.1)
LIT	593	587	6 (-61; 73)	1 (-10.4; 12.4)
NET	14,279	17,531	-3252 (-4064; -2439)	-18.5 (-23.2; -13.9)
POL	3,529	3,037	492 (229; 754)	16.2 (7.5; 24.8)
SER	2,173	2,477	-304 (-487; -120)	-12.3 (-19.7; -4.8)
SLO	459	395	64 (5; 122)	16.2 (1.3; 30.9)
SPA	36,393	38,948	-2555 (-3968; -1141)	-6.6 (-10.2; -2.9)
SWI	6,524	6,789	-265 (-550; 20)	-3.9 (-8.1; 0.3)
UK	78,386	89,781	-11,395 (-15,196; -7593)	-12.7 (-16.9; -8.5)
MAU	9[a]	.	.	.
ARG	3,091	3,478	-387 (-628; -145)	-11.1 (-18.1; -4.2)
BRA	26,737	27,801	-1064 (-2959; 831)	-3.8 (-10.6; 3.0)
CHI	4,353	3,738	615 (347; 882)	16.5 (9.3; 23.6)
CUB	5,436	5,924	-488 (-872; -103)	-8.2 (-14.7; -1.7)
GUA	207	233	-26 (-81; 29)	-11.2 (-34.8; 12.4)
MEX	3,693	3,592	101 (-305; 507)	2.8 (-8.5; 14.1)
USA	259,513	244,482	15,031 (2876; 27,185)	6.1 (1.2; 11.1)
ISR	2,777	3,084	-307 (-505; -108)	-10 (-16.4; -3.5)
JAP	41,678	50,389	-8711 (-10,615; -6806)	-17.3 (-21.1; -13.5)
KOR	10,640	10,850	-210 (-871; 451)	-1.9 (-8.0; 4.2)
AUS	14,575	15,863	-1288 (-2003; -572)	-8.1 (-12.6; -3.6)
All countries	637,112	663,616	-26,504 (-60,858; 7850)	-4.0 (-9.2; 1.2)

[a] Expected deaths were not estimated when observed deaths were <10

Table 5. Absolute and percent differences in the number of deaths from transport accidents registered in 2020 relative to the expected deaths, by country.

Country	Observed deaths	Expected deaths	Absolute difference (95% CI)	Percent difference (95% CI)
AU	369	386	-17 (-84; 50)	-4.4 (-21.8; 13.0)
BUL	439	578	-139 (-229; -48)	-24 (-39.6; -8.3)
CZE	666	684	-18 (-133; 97)	-2.6 (-19.4; 14.2)
DEN	200	214	-14 (-53; 25)	-6.5 (-24.8; 11.7)
EST	70	57	13 (-2; 28)	22.8 (-3.5; 49.1)
FIN	269	275	-6 (-54; 42)	-2.2 (-19.6; 15.3)
GEO	554	912	-358 (-567; -148)	-39.3 (-62.2; -16.2)
GER	3,118	3,314	-196 (-682; 290)	-5.9 (-20.6; 8.8)
ITA	2,618	3,321	-703 (-1181; -224)	-21.2 (-35.6; -6.7)
LAT	155	166	-11 (-42; 20)	-6.6 (-25.3; 12.0)
LIT	217	202	15 (-19; 49)	7.4 (-9.4; 24.3)
NET	669	695	-26 (-125; 73)	-3.7 (-18.0; 10.5)
POL	3,643	3,324	319 (-174; 812)	9.6 (-5.2; 24.4)
SER	495	496	-1 (-77; 75)	-0.2 (-15.5; 15.1)
SLO	122	119	3 (-22; 28)	2.5 (-18.5; 23.5)
SPA	1,726	2,057	-331 (-611; -50)	-16.1 (-29.7; -2.4)
SWI	244	249	-5 (-47; 37)	-2 (-18.9; 14.9)
UK	1,423	1,821	-398 (-696; -99)	-21.9 (-38.2; -5.4)
MAU	150	168	-18 (-56; 20)	-10.7 (-33.3; 11.9)
ARG	2,349	3,840	-1491 (-2222; -759)	-38.8 (-57.9; -19.8)
BRA	33,657	31,805	1852 (-3782; 7486)	5.8 (-11.9; 23.5)
CHI	1,749	1,873	-124 (-396; 148)	-6.6 (-21.1; 7.9)
CUB	676	984	-308 (-451; -164)	-31.3 (-45.8; -16.7)
GUA	1,345	1,908	-563 (-972; -153)	-29.5 (-50.9; -8.0)
MEX	13,561	14,986	-1425 (-4020; 1170)	-9.5 (-26.8; 7.8)
USA	45,159	43,726	1433 (-5011; 7877)	3.3 (-11.5; 18.0)
ISR	347	371	-24 (-95; 47)	-6.5 (-25.6; 12.7)
JAP	3,821	4,264	-443 (-831; -54)	-10.4 (-19.5; -1.3)
KOR	3,987	4,233	-246 (-617; 125)	-5.8 (-14.6; 3.0)
AUS	1,355	1,412	-57 (-267; 153)	-4 (-18.9; 10.8)
All countries	*125,153*	*128,486*	*-3333 (-7568; 902)*	*-2.6 (-5.9; 0.7)*

Table 6. Absolute and percent differences in the number of deaths from suicides registered in 2020 relative to the expected deaths, by country.

Country	Observed deaths	Expected deaths	Absolute difference (95% CI)	Percent difference (95% CI)
AU	1,072	1,142	-70 (-214; 74)	-6.1 (-18.7; 6.5)
BUL	588	582	6 (-74; 86)	1 (-12.7; 14.8)
CZE	1,224	1,207	17 (-167; 201)	1.4 (-13.8; 16.7)
DEN	579	575	4 (-82; 90)	0.7 (-14.3; 15.7)
EST	210	192	18 (-20; 56)	9.4 (-10.4; 29.2)
FIN	717	734	-17 (-152; 118)	-2.3 (-20.7; 16.1)
GEO	202	296	-94 (-155; -32)	-31.8 (-52.4; -10.8)
GER	9,221	9,138	83 (-949; 1115)	0.9 (-10.4; 12.2)
ITA	3,618	3,617	1 (-456; 458)	0 (-12.6; 12.7)
LAT	298	286	12 (-43; 67)	4.2 (-15.0; 23.4)
LIT	608	652	-44 (-156; 68)	-6.7 (-23.9; 10.4)
NET	1,824	1,913	-89 (-370; 192)	-4.7 (-19.3; 10.0)
POL	4,559	4,094	465 (-158; 1088)	11.4 (-3.9; 26.6)
SER	895	871	24 (-95; 143)	2.8 (-10.9; 16.4)
SLO	368	370	-2 (-59; 55)	-0.5 (-15.9; 14.9)
SPA	3,947	3,730	217 (-270; 704)	5.8 (-7.2; 18.9)
SWI	972	1,010	-38 (-168; 92)	-3.8 (-16.6; 9.1)
UK	5,527	5,501	26 (-911; 963)	0.5 (-16.6; 17.5)
MAU	135	110	25 (-6; 56)	22.7 (-5.5; 50.9)
ARG	2,803	3,261	-458 (-1176; 260)	-14 (-36.1; 8.0)
BRA	13,816	13,769	47 (-2409; 2503)	0.3 (-17.5; 18.2)
CHI	1,602	1,835	-233 (-574; 108)	-12.7 (-31.3; 5.9)
CUB	1,556	1,473	83 (-110; 276)	5.6 (-7.5; 18.7)
GUA	574	674	-100 (-299; 99)	-14.8 (-44.4; 14.7)
MEX	7,739	7,134	605 (-968; 2178)	8.5 (-13.6; 30.5)
USA	45,977	49,960	-3983 (-11,872; 3906)	-8 (-23.8; 7.8)
ISR	437	417	20 (-74; 114)	4.8 (-17.7; 27.3)
JAP	20,212	17,786	2426 (-76; 4928)	13.6 (-0.4; 27.7)
KOR	13,196	12,646	550 (-1280; 2380)	4.3 (-10.1; 18.8)
AUS	3,139	3,558	-419 (-1014; 176)	-11.8 (-28.5; 4.9)
All countries	*147,615*	*148,5747*	*-1132 (-13,427; 11,163)*	*-0.8 (-9.0; 7.5)*

Table 7. Absolute and percent differences in the number of deaths from ill-defined causes registered in 2020 relative to the expected deaths, by country.

**Country**	**Observed deaths**	**Expected deaths**	**Absolute difference (95% CI)**	**Percent difference (95% CI)**
AU	3,074	2,836	238 (74; 401)	8.4 (2.6; 14.1)
BUL	4,752	4,232	520 (181; 858)	12.3 (4.3; 20.3)
CZE	3,676	1,992	1684 (1523; 1844)	84.5 (76.5; 92.6)
DEN	4,525	3,969	556 (203; 908)	14 (5.1; 22.9)
EST	237	330	-93 (-148; -37)	-28.2 (-44.8; -11.2)
FIN	332	356	-24 (-87; 39)	-6.7 (-24.4; 11.0)
GEO	6,718	7,812	-1094 (-2059; -128)	-14 (-26.4; -1.6)
GER	35,145	37,496	-2351 (-4291; -410)	-6.3 (-11.4; -1.1)
ITA	25,932	15,767	10,165 (9465; 10,864)	64.5 (60.0; 68.9)
LAT	326	167	159 (113; 204)	95.2 (67.7; 122.2)
LIT	741	654	87 (-15; 189)	13.3 (-2.3; 28.9)
NET	8,994	7,324	1670 (1290; 2049)	22.8 (17.6; 28.0)
POL	49,695	47,525	2170 (-1367; 5707)	4.6 (-2.9; 12.0)
SER	9,687	6,153	3534 (3027; 4040)	57.4 (49.2; 65.7)
SLO	611	709	-98 (-177; -18)	-13.8 (-25.0; -2.5)
SPA	9,420	9,144	276 (-284; 836)	3 (-3.1; 9.1)
SWI	4,290	3,955	335 (97; 572)	8.5 (2.5; 14.5)
UK	17,942	15,238	2704 (1915; 3492)	17.7 (12.6; 22.9)
MAU	514	128	386 (361; 410)	301.6 (282.0; 320.3)
ARG	23,315	19,940	3375 (2159; 4590)	16.9 (10.8; 23.0)
BRA	89,629	70,680	18,949 (13,683; 24,214)	26.8 (19.4; 34.3)
CHI	2,920	2,703	217 (-46; 480)	8 (-1.7; 17.8)
CUB	1,091	1,084	7 (-103; 117)	0.6 (-9.5; 10.8)
GUA	13,199	12,738	461 (-1140; 2062)	3.6 (-8.9; 16.2)
MEX	10,189	8,185	2004 (1344; 2663)	24.5 (16.4; 32.5)
USA	48,711	34,824	13,887 (9207; 18,566)	39.9 (26.4; 53.3)
ISR	2,914	2,979	-65 (-321; 191)	-2.2 (-10.8; 6.4)
JAP	159,805	161,548	-1743 (-5892; 2406)	-1.1 (-3.6; 1.5)
KOR	33,883	27,791	6092 (4777; 7406)	21.9 (17.2; 26.6)
AUS	2,988	1,768	1220 (980; 1459)	69 (55.4; 82.5)
All countries	*575,255*	*510,154*	*65,101 (9381; 120,820)*	*12.8 (1.8; 23.7)*

Table 8. Absolute and percent differences in the number of deaths from any cause registered in 2020 relative to the expected deaths and COVID-19 deaths, by sex and age group (<75 vs ≥75 years) in countries with populations of ≥10 million.

Sex/Age group	Country	Observed deaths	Expected deaths	Absolute difference (95% CI)	Percent difference (95% CI)	COVID-19 deaths	COVID-19 mortality rate (per 10,000)
M, age<75	CZE	32,711	29,586	3125 (2773; 3476)	10.6 (9.4; 11.7)	3,429	6.7
F, age<75	CZE	17,379	16,136	1243 (995; 1490)	7.7 (6.2; 9.2)	1,799	3.5
M, age≥75	CZE	33,888	27,466	6422 (6084; 6759)	23.4 (22.2; 24.6)	2,509	166.5
F, age≥75	CZE	45,311	38,836	6475 (5960; 6989)	16.7 (15.3; 18.0)	2,802	95.4
M, age<75	GER	180,700	179,575	1125 (-1656; 3906)	0.6 (-0.9; 2.2)	8,077	2.1
F, age<75	GER	103,147	103,372	-225 (-1830; 1380)	-0.2 (-1.8; 1.3)	3,898	1.0
M, age≥75	GER	312,097	305,099	6998 (2618; 11,377)	2.3 (0.9; 3.7)	12,863	57.9
F, age≥75	GER	389,628	387,361	2267 (-2445; 6979)	0.6 (-0.6; 1.8)	14,920	41.6
M, age<75	ITA	111,240	95,725	15,515 (14,422; 16,607)	16.2 (15.1; 17.3)	20,606	7.5
F, age<75	ITA	64,012	59,228	4784 (3859; 5708)	8.1 (6.5; 9.6)	8,459	3.0
M, age≥75	ITA	248,694	211,005	37,689 (35,569; 39,808)	17.9 (16.9; 18.9)	23,286	140.7
F, age≥75	ITA	316,497	275,288	41,209 (38,053; 44,364)	15.0 (13.8; 16.1)	25,790	93.3
M, age<75	NET	31,328	28,910	2418 (2019; 2816)	8.4 (7.0; 9.7)	4,335	5.2
F, age<75	NET	21,684	21,175	509 (110; 907)	2.4 (0.5; 4.3)	2,411	2.9
M, age≥75	NET	52,989	46,659	6330 (5729; 6930)	13.6 (12.3; 14.9)	6,446	185.2
F, age≥75	NET	62,677	56,372	6305 (5420; 7189)	11.2 (9.6; 12.8)	6,981	133.1
M, age<75	POL	146,021	125,568	20,453 (19,134; 21,771)	16.3 (15.2; 17.3)	15,583	8.7
F, age<75	POL	72,879	64,027	8852 (8075; 9628)	13.8 (12.6; 15.0)	8,284	4.5
M, age≥75	POL	103,723	84,650	19,073 (18,100; 20,045)	22.5 (21.4; 23.7)	8,383	157.4
F, age≥75	POL	154,732	134,293	20,439 (19,005; 21,872)	15.2 (14.2; 16.3)	9,201	78.4
M, age<75	SPA	86,930	78,623	8307 (7277; 9336)	10.6 (9.3; 11.9)	15,574	7.0
F, age<75	SPA	43,602	38,702	4900 (4271; 5528)	12.7 (11.0; 14.3)	7,441	3.3
M, age≥75	SPA	162,734	136,364	26,370 (24,723; 28,016)	19.3 (18.1; 20.5)	23,343	220.2
F, age≥75	SPA	200,510	171,028	29,482 (27,422; 31,541)	17.2 (16.0; 18.4)	28,481	158.7
M, age<75	UK	131,409	118,786	12,623 (11,193; 14,052)	10.6 (9.4; 11.8)	19,902	6.3
F, age<75	UK	87,724	83,183	4541 (3345; 5736)	5.5 (4.0; 6.9)	11,395	3.6
M, age≥75	UK	217,025	192,882	24,143 (21,969; 26,316)	12.5 (11.4; 13.6)	24,934	181.3
F, age≥75	UK	252,647	232,986	19,661 (16,871; 22,450)	8.4 (7.2; 9.6)	25,123	125.1
M, age<75	ARG	109,144	100,072	9072 (7765; 10,378)	9.1 (7.8; 10.4)	21,737	10.0
F, age<75	ARG	67,217	62,557	4660 (3794; 5525)	7.4 (6.1; 8.8)	12,219	5.5
M, age≥75	ARG	84,669	75,936	8733 (7671; 9794)	11.5 (10.1; 12.9)	8,117	209.3
F, age≥75	ARG	109,383	105,705	3678 (2323; 5032)	3.5 (2.2; 4.8)	10,271	127.8
M, age<75	BRA	577,920	503,296	74,624 (69,605; 79,642)	14.8 (13.8; 15.8)	91,711	8.9
F, age<75	BRA	345,085	298,637	46,448 (44,394; 48,501)	15.6 (14.9; 16.2)	61,084	5.8
M, age≥75	BRA	294,414	256,770	37,644 (34,665; 40,622)	14.7 (13.5; 15.8)	29,910	193.1
F, age≥75	BRA	336,722	316,358	20,364 (18,164; 22,563)	6.4 (5.7; 7.1)	29,966	114.8
M, age<75	CHI	36,859	31,811	5048 (4628; 5467)	15.9 (14.5; 17.2)	7,546	8.2
F, age<75	CHI	21,998	19,577	2421 (2200; 2641)	12.4 (11.2; 13.5)	4,359	4.7
M, age≥75	CHI	30,772	26,068	4704 (4340; 5067)	18.0 (16.6; 19.4)	3,249	166.7
F, age≥75	CHI	36,521	32,484	4037 (3697; 4376)	12.4 (11.4; 13.5)	3,526	102.9
M, age<75	CUB	31,338	31,274	64 (-349; 477)	0.2 (-1.1; 1.5)	55	0.1
F, age<75	CUB	20,194	20,026	168 (-117; 453)	0.8 (-0.6; 2.3)	33	0.1
M, age≥75	CUB	30,153	30,714	-561 (-975; -146)	-1.8 (-3.2; -0.5)	30	1.7
F, age≥75	CUB	30,699	31,122	-423 (-838; -7)	-1.4 (-2.7; 0.0)	25	1.0

Sex/Age group	Country	Observed deaths	Expected deaths	Absolute difference (95% CI)	Percent difference (95% CI)	COVID-19 deaths	COVID-19 mortality rate (per 10,000)
M, age<75	GUA	38,813	34,954	3859 (3191; 4526)	11.0 (9.1; 12.9)	5,042	5.8
F, age<75	GUA	25,929	25,154	775 (407; 1142)	3.1 (1.6; 4.5)	2,047	2.3
M, age≥75	GUA	16,142	13,014	3128 (2819; 3436)	24.0 (21.7; 26.4)	592	70.3
F, age≥75	GUA	14,776	14,224	552 (315; 788)	3.9 (2.2; 5.5)	299	27.2
M, age<75	MEX	435,769	280,149	155,620 (151,288; 159,951)	55.5 (54.0; 57.1)	114,047	18.3
F, age<75	MEX	253,392	177,785	75,607 (73,618; 77,595)	42.5 (41.4; 43.6)	61,213	9.5
M, age≥75	MEX	190,542	137,831	52,711 (50,215; 55,206)	38.2 (36.4; 40.1)	14,695	173.4
F, age≥75	MEX	185,904	148,980	36,924 (35,168; 38,679)	24.8 (23.6; 26.0)	10,245	86.1
M, age<75	USA	946,027	788,739	157,288 (148,158; 166,417)	19.9 (18.8; 21.1)	118,839	7.5
F, age<75	USA	602,697	520,505	82,192 (77,768; 86,615)	15.8 (14.9; 16.6)	72,630	4.6
M, age≥75	USA	823,778	724,094	99,684 (90,978; 108,389)	13.8 (12.6; 15.0)	73,670	140.4
F, age≥75	USA	1,011,111	890,580	120,531 (113,540; 127,521)	13.5 (12.7; 14.3)	85,688	108.5
M, age<75	JAP	218,786	217,024	1762 (-625; 4149)	0.8 (-0.3; 1.9)	959	0.2
F, age<75	JAP	103,618	106,236	-2618 (-4087; -1148)	-2.5 (-3.8; -1.1)	310	0.1
M, age≥75	JAP	487,713	479,440	8273 (3653; 12892)	1.7 (0.8; 2.7)	1,135	2.7
F, age≥75	JAP	562,200	566,077	-3877 (-9455; 1701)	-0.7 (-1.7; 0.3)	1,062	1.4
M, age<75	KOR	80,497	81,029	-532 (-1498; 434)	-0.7 (-1.8; 0.5)	277	0.1
F, age<75	KOR	34,260	34,905	-645 (-1295; 5)	-1.8 (-3.7; 0.0)	155	0.1
M, age≥75	KOR	84,657	81,455	3202 (2210; 4193)	3.9 (2.7; 5.1)	206	3.3
F, age≥75	KOR	105,507	104,756	751 (-868; 2370)	0.7 (-0.8; 2.3)	312	2.5
M, age<75	AUS	34,026	34,826	-800 (-1222; -377)	-2.3 (-3.5; -1.1)	142	0.1
F, age<75	AUS	20,901	22,210	-1309 (-1569; -1048)	-5.9 (-7.1; -4.7)	72	0.1
M, age≥75	AUS	50,556	53,322	-2766 (-3377; -2154)	-5.2 (-6.3; -4.0)	297	6.8
F, age≥75	AUS	55,810	58,925	-3115 (-3695; -2534)	-5.3 (-6.3; -4.3)	388	6.4

Table 9. Absolute and percent differences in the number of deaths from neoplasms registered in 2020 relative to the expected deaths, by age group (<75 vs ≥75 years) in countries with population of ≥10 million.

Sex/Age group	Country	Observed deaths	Expected deaths	Absolute difference (95% CI)	Percent difference (95% CI)
M, age<75	CZE	9,521	9,671	-150 (-474; 174)	-1.6 (-4.9; 1.8)
F, age<75	CZE	6,741	6,766	-25 (-334; 284)	-0.4 (-4.9; 4.2)
M, age≥75	CZE	6,380	5,959	421 (200; 641)	7.1 (3.4; 10.8)
F, age≥75	CZE	6,074	5,926	148 (-133; 429)	2.5 (-2.2; 7.2)
M, age<75	GER	59,847	61,882	-2035 (-3766; -303)	-3.3 (-6.1; -0.5)
F, age<75	GER	45,341	47,319	-1978 (-3919; -36)	-4.2 (-8.3; -0.1)
M, age≥75	GER	70,069	68,968	1101 (-776; 2978)	1.6 (-1.1; 4.3)
F, age≥75	GER	64,295	64,237	58 (-2430; 2546)	0.1 (-3.8; 4.0)
M, age<75	ITA	40,886	42,514	-1628 (-2742; -513)	-3.8 (-6.4; -1.2)
F, age<75	ITA	31,320	31,960	-640 (-1951; 671)	-2.0 (-6.1; 2.1)
M, age≥75	ITA	56,219	56,303	-84 (-1490; 1322)	-0.1 (-2.6; 2.3)
F, age≥75	ITA	48,013	48,033	-20 (-1857; 1817)	0.0 (-3.9; 3.8)
M, age<75	NET	12,226	12,694	-468 (-879; -56)	-3.7 (-6.9; -0.4)
F, age<75	NET	10,559	11,017	-458 (-973; 57)	-4.2 (-8.8; 0.5)
M, age≥75	NET	13,115	12,987	128 (-293; 549)	1.0 (-2.3; 4.2)
F, age≥75	NET	11,189	10,540	649 (150; 1147)	6.2 (1.4; 10.9)
M, age<75	POL	37,766	39,724	-1958 (-3339; -576)	-4.9 (-8.4; -1.5)
F, age<75	POL	28,333	30,028	-1695 (-3096; -293)	-5.6 (-10.3; -1.0)
M, age≥75	POL	21,242	20,243	999 (207; 1790)	4.9 (1.0; 8.8)
F, age≥75	POL	21,361	21,122	239 (-805; 1283)	1.1 (-3.8; 6.1)
M, age<75	SPA	32,877	34,048	-1171 (-2131; -210)	-3.4 (-6.3; -0.6)
F, age<75	SPA	20,036	19,710	326 (-579; 1231)	1.7 (-2.9; 6.2)
M, age≥75	SPA	34,370	33,898	472 (-491; 1435)	1.4 (-1.4; 4.2)
F, age≥75	SPA	25,458	25,390	68 (-1053; 1189)	0.3 (-4.1; 4.7)
M, age<75	UK	41,072	42,600	-1528 (-2984; -71)	-3.6 (-7.0; -0.2)
F, age<75	UK	35,917	37,157	-1240 (-3073; 593)	-3.3 (-8.3; 1.6)
M, age≥75	UK	50,851	50,512	339 (-1355; 2033)	0.7 (-2.7; 4.0)
F, age≥75	UK	44,461	44,155	306 (-1834; 2446)	0.7 (-4.2; 5.5)
M, age<75	ARG	19,735	21,557	-1822 (-2874; -769)	-8.5 (-13.3; -3.6)
F, age<75	ARG	18,846	19,425	-579 (-2297; 1139)	-3.0 (-11.8; 5.9)
M, age≥75	ARG	11,365	12,317	-952 (-1617; -286)	-7.7 (-13.1; -2.3)
F, age≥75	ARG	11,116	12,628	-1512 (-2719; -304)	-12.0 (-21.5; -2.4)
M, age<75	BRA	79,080	83,901	-4821 (-8669; -972)	-5.7 (-10.3; -1.2)
F, age<75	BRA	75,174	77,849	-2675 (-9378; 4028)	-3.4 (-12.0; 5.2)
M, age≥75	BRA	40,129	42,442	-2313 (-4523; -102)	-5.4 (-10.7; -0.2)
F, age≥75	BRA	34,903	37,862	-2959 (-6700; 782)	-7.8 (-17.7; 2.1)
M, age<75	CHI	8,130	8,510	-380 (-708; -51)	-4.5 (-8.3; -0.6)
F, age<75	CHI	7,632	7,765	-133 (-604; 338)	-1.7 (-7.8; 4.4)
M, age≥75	CHI	6,768	6,589	179 (-88; 446)	2.7 (-1.3; 6.8)
F, age≥75	CHI	6,126	6,013	113 (-269; 495)	1.9 (-4.5; 8.2)
M, age<75	CUB	8,824	8,813	11 (-258; 280)	0.1 (-2.9; 3.2)
F, age<75	CUB	7,000	6,891	109 (-282; 500)	1.6 (-4.1; 7.3)
M, age≥75	CUB	6,806	6,711	95 (-121; 311)	1.4 (-1.8; 4.6)
F, age≥75	CUB	4,564	4,629	-65 (-348; 218)	-1.4 (-7.5; 4.7)
M, age<75	GUA	2,672	2,535	137 (-81; 355)	5.4 (-3.2; 14.0)
F, age<75	GUA	3,613	3,465	148 (-288; 584)	4.3 (-8.3; 16.9)
M, age≥75	GUA	1,476	1,376	100 (-31; 231)	7.3 (-2.3; 16.8)
F, age≥75	GUA	1,183	1,193	-10 (-194; 174)	-0.8 (-16.3; 14.6)
M, age<75	MEX	30,396	30,731	-335 (-1969; 1299)	-1.1 (-6.4; 4.2)
F, age<75	MEX	35,311	35,974	-663 (-3869; 2543)	-1.8 (-10.8; 7.1)
M, age≥75	MEX	16,617	16,306	311 (-665; 1287)	1.9 (-4.1; 7.9)
F, age≥75	MEX	13,137	13,438	-301 (-1754; 1152)	-2.2 (-13.1; 8.6)
M, age<75	USA	183,942	187,094	-3152 (-10286; 3982)	-1.7 (-5.5; 2.1)
F, age<75	USA	159,022	161,177	-2155 (-10947; 6637)	-1.3 (-6.8; 4.1)
M, age≥75	USA	142,480	140,243	2237 (-3430; 7904)	1.6 (-2.4; 5.6)
F, age≥75	USA	133,176	133,315	-139 (-7739; 7461)	-0.1 (-5.8; 5.6)
M, age<75	JAP	86,569	89,442	-2873 (-5159; -586)	-3.2 (-5.8; -0.7)
F, age<75	JAP	52,591	53,336	-745 (-2983; 1493)	-1.4 (-5.6; 2.8)

Sex/Age group	Country	Observed deaths	Expected deaths	Absolute difference (95% CI)	Percent difference (95% CI)
M, age≥75	JAP	141,458	135,923	5535 (2300; 8769)	4.1 (1.7; 6.5)
F, age≥75	JAP	110,903	109,473	1430 (-2628; 5488)	1.3 (-2.4; 5.0)
M, age<75	KOR	28,146	29,165	-1019 (-2202; 164)	-3.5 (-7.6; 0.6)
F, age<75	KOR	14,921	15,201	-280 (-1259; 699)	-1.8 (-8.3; 4.6)
M, age≥75	KOR	23,538	22,112	1426 (485; 2366)	6.4 (2.2; 10.7)
F, age≥75	KOR	17,166	17,059	107 (-966; 1180)	0.6 (-5.7; 6.9)
M, age<75	AUS	12,631	13,256	-625 (-1127; -122)	-4.7 (-8.5; -0.9)
F, age<75	AUS	9,875	10,540	-665 (-1241; -88)	-6.3 (-11.8; -0.8)
M, age≥75	AUS	14,773	14,832	-59 (-615; 497)	-0.4 (-4.1; 3.4)
F, age≥75	AUS	11,509	11,350	159 (-459; 777)	1.4 (-4.0; 6.8)

Table 10. Absolute and percent differences in the number of deaths from cardiovascular diseases registered in 2020 relative to the expected deaths, by age group (<75 vs ≥75 years) in countries with population ≥10 million.

Sex/Age group	Country	Observed deaths	Expected deaths	Absolute difference (95% CI)	Percent difference (95% CI)
M, age<75	CZE	9,691	9,053	638 (323; 952)	7.0 (3.6; 10.5)
F, age<75	CZE	4,201	4,158	43 (-95; 181)	1.0 (-2.3; 4.4)
M, age≥75	CZE	14,142	12,760	1382 (958; 1805)	10.8 (7.5; 14.1)
F, age≥75	CZE	22,310	20,185	2125 (1626; 2623)	10.5 (8.1; 13.0)
M, age<75	GER	43,001	42,227	774 (-788; 2336)	1.8 (-1.9; 5.5)
F, age<75	GER	18,182	17,996	186 (-404; 776)	1.0 (-2.2; 4.3)
M, age≥75	GER	114,555	116,852	-2297 (-5899; 1305)	-2.0 (-5.0; 1.1)
F, age≥75	GER	162,263	165,456	-3193 (-6566; 180)	-1.9 (-4.0; 0.1)
M, age<75	ITA	22,143	21,650	493 (-297; 1283)	2.3 (-1.4; 5.9)
F, age<75	ITA	9,983	10,246	-263 (-579; 53)	-2.6 (-5.7; 0.5)
M, age≥75	ITA	75,787	75,019	768 (-1422; 2958)	1.0 (-1.9; 3.9)
F, age≥75	ITA	117,331	113,891	3440 (1416; 5463)	3.0 (1.2; 4.8)
M, age<75	NET	5,358	5,457	-99 (-312; 114)	-1.8 (-5.7; 2.1)
F, age<75	NET	2,699	2,843	-144 (-252; -35)	-5.1 (-8.9; -1.2)
M, age≥75	NET	12,038	12,326	-288 (-709; 133)	-2.3 (-5.8; 1.1)
F, age≥75	NET	15,186	15,742	-556 (-977; -134)	-3.5 (-6.2; -0.9)
M, age<75	POL	39,246	35,846	3400 (1564; 5235)	9.5 (4.4; 14.6)
F, age<75	POL	17,203	16,237	966 (443; 1488)	5.9 (2.7; 9.2)
M, age≥75	POL	42,156	37,923	4233 (2318; 6147)	11.2 (6.1; 16.2)
F, age≥75	POL	75,794	70,481	5313 (3623; 7002)	7.5 (5.1; 9.9)
M, age<75	SPA	17,297	16,236	1061 (416; 1705)	6.5 (2.6; 10.5)
F, age<75	SPA	6,597	6,161	436 (238; 633)	7.1 (3.9; 10.3)
M, age≥75	SPA	38,649	38,678	-29 (-1349; 1291)	-0.1 (-3.5; 3.3)
F, age≥75	SPA	57,310	56,090	1220 (122; 2317)	2.2 (0.2; 4.1)
M, age<75	UK	31,031	27,413	3618 (2301; 4934)	13.2 (8.4; 18.0)
F, age<75	UK	14,190	12,599	1591 (1113; 2068)	12.6 (8.8; 16.4)
M, age≥75	UK	49,966	50,944	-978 (-3199; 1243)	-1.9 (-6.3; 2.4)
F, age≥75	UK	56,454	56,799	-345 (-1969; 1279)	-0.6 (-3.5; 2.3)
M, age<75	ARG	23,164	23,740	-576 (-1956; 804)	-2.4 (-8.2; 3.4)
F, age<75	ARG	12,154	12,175	-21 (-556; 514)	-0.2 (-4.6; 4.2)
M, age≥75	ARG	24,624	24,517	107 (-1313; 1527)	0.4 (-5.4; 6.2)
F, age≥75	ARG	35,722	35,719	3 (-1289; 1295)	0.0 (-3.6; 3.6)
M, age<75	BRA	109,960	112,120	-2160 (-10318; 5998)	-1.9 (-9.2; 5.3)
F, age<75	BRA	71,690	73,055	-1365 (-5946; 3216)	-1.9 (-8.1; 4.4)
M, age≥75	BRA	79,135	81,951	-2816 (-9111; 3479)	-3.4 (-11.1; 4.2)
F, age≥75	BRA	96,765	101,469	-4704 (-10713; 1305)	-4.6 (-10.6; 1.3)
M, age<75	CHI	6,819	6,950	-131 (-546; 284)	-1.9 (-7.9; 4.1)
F, age<75	CHI	3,564	3,666	-102 (-261; 57)	-2.8 (-7.1; 1.6)
M, age≥75	CHI	7,864	7,657	207 (-244; 658)	2.7 (-3.2; 8.6)
F, age≥75	CHI	10,771	10,248	523 (153; 892)	5.1 (1.5; 8.7)
M, age<75	CUB	10,560	9,889	671 (201; 1140)	6.8 (2.0; 11.5)
F, age<75	CUB	6,508	6,007	501 (292; 709)	8.3 (4.9; 11.8)
M, age≥75	CUB	12,972	12,260	712 (148; 1275)	5.8 (1.2; 10.4)
F, age≥75	CUB	14,336	13,481	855 (448; 1261)	6.3 (3.3; 9.4)
M, age<75	GUA	4,396	3,358	1038 (703; 1372)	30.9 (20.9; 40.9)
F, age<75	GUA	3,236	2,898	338 (107; 568)	11.7 (3.7; 19.6)
M, age≥75	GUA	4,552	3,631	921 (568; 1273)	25.4 (15.6; 35.1)
F, age≥75	GUA	4,268	3,924	344 (49; 638)	8.8 (1.2; 16.3)
M, age<75	MEX	71,563	53,507	18056 (13708; 22403)	33.7 (25.6; 41.9)
F, age<75	MEX	40,795	33,434	7361 (5663; 9058)	22.0 (16.9; 27.1)
M, age≥75	MEX	68,256	51,481	16775 (12561; 20988)	32.6 (24.4; 40.8)
F, age≥75	MEX	74,177	60,026	14151 (11413; 16888)	23.6 (19.0; 28.1)
M, age<75	USA	224,638	207,196	17442 (5146; 29737)	8.4 (2.5; 14.4)
F, age<75	USA	120,446	110,842	9604 (4472; 14735)	8.7 (4.0; 13.3)
M, age≥75	USA	253,942	252,175	1767 (-12775; 16309)	0.7 (-5.1; 6.5)
F, age≥75	USA	313,627	311,310	2317 (-9658; 14292)	0.7 (-3.1; 4.6)
M, age<75	JAP	51,020	47,107	3913 (1924; 5901)	8.3 (4.1; 12.5)
F, age<75	JAP	18,689	18,362	327 (-205; 859)	1.8 (-1.1; 4.7)

Sex/Age group	Country	Observed deaths	Expected deaths	Absolute difference (95% CI)	Percent difference (95% CI)
M, age≥75	JAP	115,820	115,463	357 (-3812; 4526)	0.3 (-3.3; 3.9)
F, age≥75	JAP	159,251	161,505	-2254 (-5210; 702)	-1.4 (-3.2; 0.4)
M, age<75	KOR	12,312	12,544	-232 (-977; 513)	-1.8 (-7.8; 4.1)
F, age<75	KOR	4,754	5,361	-607 (-834; -379)	-11.3 (-15.6; -7.1)
M, age≥75	KOR	16,182	16,872	-690 (-1642; 262)	-4.1 (-9.7; 1.6)
F, age≥75	KOR	26,794	27,539	-745 (-1601; 111)	-2.7 (-5.8; 0.4)
M, age<75	AUS	6,696	6,630	66 (-307; 439)	1.0 (-4.6; 6.6)
F, age<75	AUS	2,903	2,920	-17 (-145; 111)	-0.6 (-5.0; 3.8)
M, age≥75	AUS	13,727	15,088	-1361 (-2101; -620)	-9.0 (-13.9; -4.1)
F, age≥75	AUS	16,962	18,252	-1290 (-1836; -743)	-7.1 (-10.1; -4.1)

Table 11. Absolute and percent differences in the number of deaths from ischemic heart diseases registered in 2020 relative to the expected deaths, by age group (<75 vs ≥75 years) in countries with population of ≥10 million.

Sex/Age group	Country	Observed deaths	Expected deaths	Absolute difference (95% CI)	Percent difference (95% CI)
M, age<75	CZE	4,693	4,538	155 (-11; 321)	3.4 (-0.2; 7.1)
F, age<75	CZE	1,576	1,635	-59 (-120; 2)	-3.6 (-7.3; 0.1)
M, age≥75	CZE	7,152	6,525	627 (399; 854)	9.6 (6.1; 13.1)
F, age≥75	CZE	9,932	9,302	630 (381; 878)	6.8 (4.1; 9.4)
M, age<75	GER	21,183	20,508	675 (-133; 1483)	3.3 (-0.6; 7.2)
F, age<75	GER	6,241	5,902	339 (137; 540)	5.7 (2.3; 9.1)
M, age≥75	GER	47,416	48,338	-922 (-2558; 714)	-1.9 (-5.3; 1.5)
F, age≥75	GER	46,622	47,469	-847 (-1898; 204)	-1.8 (-4.0; 0.4)
M, age<75	ITA	9,476	8,691	785 (448; 1121)	9.0 (5.2; 12.9)
F, age<75	ITA	2,887	2,575	312 (232; 391)	12.1 (9.0; 15.2)
M, age≥75	ITA	24,299	23,402	897 (130; 1663)	3.8 (0.6; 7.1)
F, age≥75	ITA	26,694	25,546	1148 (664; 1631)	4.5 (2.6; 6.4)
M, age<75	NET	1,935	1,833	102 (7; 196)	5.6 (0.4; 10.7)
F, age<75	NET	676	619	57 (26; 87)	9.2 (4.2; 14.1)
M, age≥75	NET	3,021	2,946	75 (-66; 216)	2.5 (-2.2; 7.3)
F, age≥75	NET	2,405	2,410	-5 (-97; 87)	-0.2 (-4.0; 3.6)
M, age<75	POL	15,105	12,037	3068 (2435; 3700)	25.5 (20.2; 30.7)
F, age<75	POL	5,465	4,549	916 (683; 1148)	20.1 (15.0; 25.2)
M, age≥75	POL	13,381	9,796	3585 (3054; 4115)	36.6 (31.2; 42.0)
F, age≥75	POL	20,452	15,390	5062 (4442; 5681)	32.9 (28.9; 36.9)
M, age<75	SPA	7,429	6,777	652 (333; 970)	9.6 (4.9; 14.3)
F, age<75	SPA	1,716	1,559	157 (92; 221)	10.1 (5.9; 14.2)
M, age≥75	SPA	10,694	10,951	-257 (-734; 220)	-2.3 (-6.7; 2.0)
F, age≥75	SPA	9,815	9,931	-116 (-399; 167)	-1.2 (-4.0; 1.7)
M, age<75	UK	18,272	15,785	2487 (1656; 3317)	15.8 (10.5; 21.0)
F, age<75	UK	5,685	4,785	900 (691; 1108)	18.8 (14.4; 23.2)
M, age≥75	UK	22,761	23,498	-737 (-1905; 431)	-3.1 (-8.1; 1.8)
F, age≥75	UK	17,332	17,809	-477 (-1096; 142)	-2.7 (-6.2; 0.8)
M, age<75	ARG	7,309	7,776	-467 (-1003; 69)	-6.0 (-12.9; 0.9)
F, age<75	ARG	2,796	2,892	-96 (-243; 51)	-3.3 (-8.4; 1.8)
M, age≥75	ARG	5,481	5,563	-82 (-489; 325)	-1.5 (-8.8; 5.8)
F, age≥75	ARG	6,934	6,576	358 (68; 647)	5.4 (1.0; 9.8)
M, age<75	BRA	42,900	46,220	-3320 (-7158; 518)	-7.2 (-15.5; 1.1)
F, age<75	BRA	21,599	23,744	-2145 (-3754; -535)	-9.0 (-15.8; -2.3)
M, age≥75	BRA	22,215	24,398	-2183 (-4462; 96)	-8.9 (-18.3; 0.4)
F, age≥75	BRA	22,776	25,923	-3147 (-4877; -1416)	-12.1 (-18.8; -5.5)
M, age<75	CHI	2,866	3,029	-163 (-382; 56)	-5.4 (-12.6; 1.8)
F, age<75	CHI	1,004	1,078	-74 (-133; -14)	-6.9 (-12.3; -1.3)
M, age≥75	CHI	2,033	2,124	-91 (-254; 72)	-4.3 (-12.0; 3.4)
F, age≥75	CHI	2,056	2,109	-53 (-157; 51)	-2.5 (-7.4; 2.4)
M, age<75	CUB	4,656	4,396	260 (27; 492)	5.9 (0.6; 11.2)
F, age<75	CUB	2,609	2,407	202 (114; 289)	8.4 (4.7; 12.0)
M, age≥75	CUB	5,414	4,981	433 (173; 692)	8.7 (3.5; 13.9)
F, age≥75	CUB	6,102	5,520	582 (406; 757)	10.5 (7.4; 13.7)
M, age<75	GUA	2,255	1,621	634 (413; 854)	39.1 (25.5; 52.7)
F, age<75	GUA	1,386	1,144	242 (123; 360)	21.2 (10.8; 31.5)
M, age≥75	GUA	2,348	1,712	636 (408; 863)	37.1 (23.8; 50.4)
F, age≥75	GUA	2,061	1,715	346 (182; 509)	20.2 (10.6; 29.7)
M, age<75	MEX	48,274	34,080	14,194 (11027; 17,360)	41.6 (32.4; 50.9)
F, age<75	MEX	23,677	17,143	6534 (5503; 7564)	38.1 (32.1; 44.1)
M, age≥75	MEX	45,896	31,728	14,168 (11,181; 17,154)	44.7 (35.2; 54.1)
F, age≥75	MEX	45,838	34,937	10,901 (9071; 12,730)	31.2 (26.0; 36.4)
M, age<75	USA	112,662	101,482	11,180 (5091; 17,268)	11.0 (5.0; 17.0)
F, age<75	USA	47,074	40,484	6590 (4731; 8448)	16.3 (11.7; 20.9)
M, age≥75	USA	115,213	112,325	2888 (-3780; 9556)	2.6 (-3.4; 8.5)
F, age≥75	USA	107,854	103,975	3879 (-196; 7954)	3.7 (-0.2; 7.6)
M, age<75	JAP	15,860	14,369	1491 (791; 2190)	10.4 (5.5; 15.2)
F, age<75	JAP	4,058	3,777	281 (162; 399)	7.4 (4.3; 10.6)

Sex/Age group	Country	Observed deaths	Expected deaths	Absolute difference (95% CI)	Percent difference (95% CI)
M, age≥75	JAP	24,318	24,108	210 (-865; 1285)	0.9 (-3.6; 5.3)
F, age≥75	JAP	23,042	23,059	-17 (-529; 495)	-0.1 (-2.3; 2.1)
M, age<75	KOR	4,096	3,900	196 (-99; 491)	5.0 (-2.5; 12.6)
F, age<75	KOR	955	995	-40 (-87; 7)	-4.0 (-8.7; 0.7)
M, age≥75	KOR	3,863	4,132	-269 (-579; 41)	-6.5 (-14.0; 1.0)
F, age≥75	KOR	5,141	5,293	-152 (-336; 32)	-2.9 (-6.3; 0.6)
M, age<75	AUS	3,759	3,650	109 (-133; 351)	3.0 (-3.6; 9.6)
F, age<75	AUS	1,015	994	21 (-30; 72)	2.1 (-3.0; 7.2)
M, age≥75	AUS	6,281	6,874	-593 (-1005; -180)	-8.6 (-14.6; -2.6)
F, age≥75	AUS	5,532	6,178	-646 (-865; -426)	-10.5 (-14.0; -6.9)

Table 12. Absolute and percent differences in the number of deaths from cerebrovascular diseases registered in 2020 relative to the expected deaths, by age group (<75 vs ≥75 years) in countries with population ≥10 million.

Sex/Age group	Country	Observed deaths	Expected deaths	Absolute difference (95% CI)	Percent difference (95% CI)
M, age<75	CZE	1,308	1,157	151 (102; 199)	13.1 (8.8; 17.2)
F, age<75	CZE	719	661	58 (30; 85)	8.8 (4.5; 12.9)
M, age≥75	CZE	2,048	2,002	46 (-31; 123)	2.3 (-1.5; 6.1)
F, age≥75	CZE	3,477	3,391	86 (-20; 192)	2.5 (-0.6; 5.7)
M, age<75	GER	6,247	5,743	504 (300; 707)	8.8 (5.2; 12.3)
F, age<75	GER	3,952	3,481	471 (346; 595)	13.5 (9.9; 17.1)
M, age≥75	GER	17,082	17,885	-803 (-1319; -286)	-4.5 (-7.4; -1.6)
F, age≥75	GER	26,027	26,723	-696 (-1324; -67)	-2.6 (-5.0; -0.3)
M, age<75	ITA	4,397	3,745	652 (522; 781)	17.4 (13.9; 20.9)
F, age<75	ITA	2,954	2,600	354 (266; 441)	13.6 (10.2; 17.0)
M, age≥75	ITA	18,513	18,123	390 (-75; 855)	2.2 (-0.4; 4.7)
F, age≥75	ITA	31,197	30,023	1174 (597; 1750)	3.9 (2.0; 5.8)
M, age<75	NET	1,089	1,091	-2 (-53; 49)	-0.2 (-4.9; 4.5)
F, age<75	NET	799	814	-15 (-53; 23)	-1.8 (-6.5; 2.8)
M, age≥75	NET	2,736	2,982	-246 (-364; -127)	-8.2 (-12.2; -4.3)
F, age≥75	NET	4,226	4,482	-256 (-404; -107)	-5.7 (-9.0; -2.4)
M, age<75	POL	7,694	6,287	1407 (1059; 1754)	22.4 (16.8; 27.9)
F, age<75	POL	4,050	3,464	586 (443; 728)	16.9 (12.8; 21.0)
M, age≥75	POL	7,052	6,035	1017 (682; 1351)	16.9 (11.3; 22.4)
F, age≥75	POL	13,744	11,434	2310 (1932; 2687)	20.2 (16.9; 23.5)
M, age<75	SPA	2,987	2,704	283 (179; 386)	10.5 (6.6; 14.3)
F, age<75	SPA	1,721	1,522	199 (143; 254)	13.1 (9.4; 16.7)
M, age≥75	SPA	8,277	8,186	91 (-166; 348)	1.1 (-2.0; 4.3)
F, age≥75	SPA	12,832	12,591	241 (-50; 532)	1.9 (-0.4; 4.2)
M, age<75	UK	4,392	3,852	540 (358; 721)	14.0 (9.3; 18.7)
F, age<75	UK	3,378	2,972	406 (282; 529)	13.7 (9.5; 17.8)
M, age≥75	UK	10,671	11,282	-611 (-1054; -167)	-5.4 (-9.3; -1.5)
F, age≥75	UK	16,019	16,371	-352 (-841; 137)	-2.2 (-5.1; 0.8)
M, age<75	ARG	4,736	5,204	-468 (-807; -128)	-9.0 (-15.5; -2.5)
F, age<75	ARG	2,912	3,206	-294 (-493; -94)	-9.2 (-15.4; -2.9)
M, age≥75	ARG	4,165	4,834	-669 (-989; -348)	-13.8 (-20.5; -7.2)
F, age≥75	ARG	5,666	6,739	-1073 (-1441; -704)	-15.9 (-21.4; -10.4)
M, age<75	BRA	27,301	26,766	535 (-1202; 2272)	2.0 (-4.5; 8.5)
F, age<75	BRA	20,444	20,268	176 (-1247; 1599)	0.9 (-6.2; 7.9)
M, age≥75	BRA	23,442	24,278	-836 (-2437; 765)	-3.4 (-10.0; 3.2)
F, age≥75	BRA	27,625	29,460	-1835 (-3776; 106)	-6.2 (-12.8; 0.4)
M, age<75	CHI	1,637	1,687	-50 (-153; 53)	-3.0 (-9.1; 3.1)
F, age<75	CHI	1,048	1,094	-46 (-112; 20)	-4.2 (-10.2; 1.8)
M, age≥75	CHI	2,250	2,205	45 (-84; 174)	2.0 (-3.8; 7.9)
F, age≥75	CHI	3,013	2,819	194 (49; 338)	6.9 (1.7; 12.0)
M, age<75	CUB	2,482	2,254	228 (117; 338)	10.1 (5.2; 15.0)
F, age<75	CUB	1,652	1,467	185 (124; 245)	12.6 (8.5; 16.7)
M, age≥75	CUB	3,203	3,109	94 (-50; 238)	3.0 (-1.6; 7.7)
F, age≥75	CUB	3,638	3,494	144 (20; 267)	4.1 (0.6; 7.6)
M, age<75	GUA	894	778	116 (34; 197)	14.9 (4.4; 25.3)
F, age<75	GUA	836	750	86 (11; 160)	11.5 (1.5; 21.3)

Sex/Age group	Country	Observed deaths	Expected deaths	Absolute difference (95% CI)	Percent difference (95% CI)
M, age≥75	GUA	969	843	126 (39; 212)	14.9 (4.6; 25.1)
F, age≥75	GUA	907	900	7 (-79; 93)	0.8 (-8.8; 10.3)
M, age<75	MEX	9,553	8,876	677 (24; 1329)	7.6 (0.3; 15.0)
F, age<75	MEX	6,754	6,927	-173 (-548; 202)	-2.5 (-7.9; 2.9)
M, age≥75	MEX	8,942	8,851	91 (-560; 742)	1.0 (-6.3; 8.4)
F, age≥75	MEX	10,933	10,858	75 (-470; 620)	0.7 (-4.3; 5.7)
M, age<75	USA	27,555	25,632	1923 (661; 3184)	7.5 (2.6; 12.4)
F, age<75	USA	21,678	20,621	1057 (176; 1937)	5.1 (0.9; 9.4)
M, age≥75	USA	42,080	40,472	1608 (-240; 3456)	4.0 (-0.6; 8.5)
F, age≥75	USA	68,949	67,267	1682 (-619; 3983)	2.5 (-0.9; 5.9)
M, age<75	JAP	15,295	13,716	1579 (919; 2238)	11.5 (6.7; 16.3)
F, age<75	JAP	6,826	6,118	708 (486; 929)	11.6 (7.9; 15.2)
M, age≥75	JAP	35,079	35,363	-284 (-1732; 1164)	-0.8 (-4.9; 3.3)
F, age≥75	JAP	45,760	46,323	-563 (-1684; 558)	-1.2 (-3.6; 1.2)
M, age<75	KOR	4,499	4,288	211 (-70; 492)	4.9 (-1.6; 11.5)
F, age<75	KOR	2,193	2,190	3 (-107; 113)	0.1 (-4.9; 5.2)
M, age≥75	KOR	6,130	6,154	-24 (-404; 356)	-0.4 (-6.6; 5.8)
F, age≥75	KOR	9,036	8,856	180 (-171; 531)	2.0 (-1.9; 6.0)
M, age<75	AUS	1,003	959	44 (-8; 96)	4.6 (-0.8; 10.0)
F, age<75	AUS	760	732	28 (-8; 64)	3.8 (-1.1; 8.7)
M, age≥75	AUS	2,971	3,181	-210 (-350; -69)	-6.6 (-11.0; -2.2)
F, age≥75	AUS	4,735	4,934	-199 (-365; -32)	-4.0 (-7.4; -0.6)

Table 13. Absolute and percent differences in the number of deaths from diabetes registered in 2020 relative to the expected deaths, by age group (<75 vs ≥75 years) in countries with population ≥10 million.

Sex/Age group	Country	Observed deaths	Expected deaths	Absolute difference (95% CI)	Percent difference (95% CI)
M, age<75	CZE	966	1,010	-44 (-109; 21)	-4.4 (-10.8; 2.1)
F, age<75	CZE	574	615	-41 (-85; 3)	-6.7 (-13.8; 0.5)
M, age≥75	CZE	1,352	1,208	144 (67; 220)	11.9 (5.5; 18.2)
F, age≥75	CZE	2,101	1,947	154 (40; 267)	7.9 (2.1; 13.7)
M, age<75	GER	3,904	3,801	103 (-44; 250)	2.7 (-1.2; 6.6)
F, age<75	GER	1,815	1,640	175 (111; 238)	10.7 (6.8; 14.5)
M, age≥75	GER	8,263	8,063	200 (-71; 471)	2.5 (-0.9; 5.8)
F, age≥75	GER	11,825	11,168	657 (366; 947)	5.9 (3.3; 8.5)
M, age<75	ITA	3,299	2,966	333 (228; 437)	11.2 (7.7; 14.7)
F, age<75	ITA	1,709	1,581	128 (63; 192)	8.1 (4.0; 12.1)
M, age≥75	ITA	8,482	7,168	1314 (1098; 1529)	18.3 (15.3; 21.3)
F, age≥75	ITA	12,023	9,911	2112 (1834; 2389)	21.3 (18.5; 24.1)
M, age<75	NET	579	530	49 (14; 83)	9.2 (2.6; 15.7)
F, age<75	NET	267	256	11 (-5; 27)	4.3 (-2.0; 10.5)
M, age≥75	NET	869	830	39 (-10; 88)	4.7 (-1.2; 10.6)
F, age≥75	NET	1,084	1,073	11 (-41; 63)	1.0 (-3.8; 5.9)
M, age<75	POL	3,142	2,596	546 (373; 718)	21.0 (14.4; 27.7)
F, age<75	POL	1,773	1,594	179 (109; 248)	11.2 (6.8; 15.6)
M, age≥75	POL	2,420	1,811	609 (481; 736)	33.6 (26.6; 40.6)
F, age≥75	POL	4,825	3,637	1188 (1053; 1322)	32.7 (29.0; 36.3)
M, age<75	SPA	1,280	1,120	160 (115; 204)	14.3 (10.3; 18.2)
F, age<75	SPA	546	507	39 (13; 64)	7.7 (2.6; 12.6)
M, age≥75	SPA	3,804	3,175	629 (523; 734)	19.8 (16.5; 23.1)
F, age≥75	SPA	5,667	4,637	1030 (888; 1171)	22.2 (19.2; 25.3)
M, age<75	UK	1,713	1,521	192 (85; 298)	12.6 (5.6; 19.6)
F, age<75	UK	986	915	71 (16; 125)	7.8 (1.7; 13.7)
M, age≥75	UK	2,892	2,763	129 (-45; 303)	4.7 (-1.6; 11.0)
F, age≥75	UK	3,311	2,999	312 (170; 453)	10.4 (5.7; 15.1)
M, age<75	ARG	3,194	3,152	42 (-163; 247)	1.3 (-5.2; 7.8)
F, age<75	ARG	2,169	2,073	96 (-23; 215)	4.6 (-1.1; 10.4)
M, age≥75	ARG	2,059	2,023	36 (-107; 179)	1.8 (-5.3; 8.8)
F, age≥75	ARG	2,378	2,333	45 (-87; 177)	1.9 (-3.7; 7.6)
M, age<75	BRA	22,139	19,144	2995 (1664; 4325)	15.6 (8.7; 22.6)
F, age<75	BRA	19,779	17,300	2479 (1573; 3384)	14.3 (9.1; 19.6)
M, age≥75	BRA	13,584	12,115	1469 (554; 2383)	12.1 (4.6; 19.7)
F, age≥75	BRA	20,202	18,520	1682 (724; 2639)	9.1 (3.9; 14.2)
M, age<75	CHI	923	962	-39 (-131; 53)	-4.1 (-13.6; 5.5)
F, age<75	CHI	631	772	-141 (-216; -65)	-18.3 (-28.0; -8.4)
M, age≥75	CHI	926	939	-13 (-103; 77)	-1.4 (-11.0; 8.2)
F, age≥75	CHI	1,185	1,254	-69 (-182; 44)	-5.5 (-14.5; 3.5)
M, age<75	CUB	742	611	131 (85; 176)	21.4 (13.9; 28.8)
F, age<75	CUB	827	674	153 (112; 193)	22.7 (16.6; 28.6)
M, age≥75	CUB	498	441	57 (21; 92)	12.9 (4.8; 20.9)
F, age≥75	CUB	820	703	117 (74; 159)	16.6 (10.5; 22.6)
M, age<75	GUA	3,427	2,107	1320 (986; 1653)	62.6 (46.8; 78.5)
F, age<75	GUA	3,962	3,028	934 (607; 1260)	30.8 (20.0; 41.6)
M, age≥75	GUA	1,090	764	326 (178; 473)	42.7 (23.3; 61.9)
F, age≥75	GUA	1,517	1,254	263 (102; 423)	21.0 (8.1; 33.7)
M, age<75	MEX	55,212	38,098	17,114 (13,566; 20,661)	44.9 (35.6; 54.2)
F, age<75	MEX	43,733	34,494	9239 (6846; 11,631)	26.8 (19.8; 33.7)
M, age≥75	MEX	22,575	16,429	6146 (4341; 7950)	37.4 (26.4; 48.4)
F, age≥75	MEX	27,076	21,107	5969 (4362; 7575)	28.3 (20.7; 35.9)
M, age<75	USA	35,152	29,712	5440 (3353; 7526)	18.3 (11.3; 25.3)
F, age<75	USA	22,054	17,870	4184 (3047; 5320)	23.4 (17.1; 29.8)
M, age≥75	USA	22,380	20,925	1455 (-121; 3031)	7.0 (-0.6; 14.5)
F, age≥75	USA	22,601	20,277	2324 (1049; 3598)	11.5 (5.2; 17.7)
M, age<75	JAP	3,046	2,936	110 (-31; 251)	3.7 (-1.1; 8.5)
F, age<75	JAP	989	958	31 (-8; 70)	3.2 (-0.8; 7.3)

Sex/Age group	Country	Observed deaths	Expected deaths	Absolute difference (95% CI)	Percent difference (95% CI)
M, age≥75	JAP	4,719	4,509	210 (8; 411)	4.7 (0.2; 9.1)
F, age≥75	JAP	5,145	4,996	149 (-2; 300)	3.0 (0.0; 6.0)
M, age<75	KOR	2,026	1,983	43 (-75; 161)	2.2 (-3.8; 8.1)
F, age<75	KOR	797	945	-148 (-205; -90)	-15.7 (-21.7; -9.5)
M, age≥75	KOR	2,294	2,085	209 (84; 333)	10.0 (4.0; 16.0)
F, age≥75	KOR	3,338	3,152	186 (28; 343)	5.9 (0.9; 10.9)
M, age<75	AUS	1,141	1,027	114 (53; 174)	11.1 (5.2; 16.9)
F, age<75	AUS	565	556	9 (-24; 42)	1.6 (-4.3; 7.6)
M, age≥75	AUS	1,729	1,784	-55 (-151; 41)	-3.1 (-8.5; 2.3)
F, age≥75	AUS	1,713	1,729	-16 (-101; 69)	-0.9 (-5.8; 4.0)

Table 14. Absolute and percent differences in the number of deaths from influenza and pneumonia registered in 2020 relative to the expected deaths, by age group (<75 vs ≥75 years) in countries with population ≥10 million.

Sex/Age group	Country	Observed deaths	Expected deaths	Absolute difference (95% CI)	Percent difference (95% CI)
M, age<75	CZE	822	775	47 (2; 91)	6.1 (0.3; 11.7)
F, age<75	CZE	354	390	-36 (-61; -10)	-9.2 (-15.6; -2.6)
M, age≥75	CZE	1,334	1,217	117 (52; 181)	9.6 (4.3; 14.9)
F, age≥75	CZE	1,421	1,526	-105 (-179; -30)	-6.9 (-11.7; -2.0)
M, age<75	GER	2,023	2,288	-265 (-372; -157)	-11.6 (-16.3; -6.9)
F, age<75	GER	954	1,159	-205 (-271; -138)	-17.7 (-23.4; -11.9)
M, age≥75	GER	7,318	8,977	-1659 (-1984; -1333)	-18.5 (-22.1; -14.8)
F, age≥75	GER	6,911	9,411	-2500 (-2843; -2156)	-26.6 (-30.2; -22.9)
M, age<75	ITA	1,306	1,078	228 (170; 285)	21.2 (15.8; 26.4)
F, age<75	ITA	600	595	5 (-33; 43)	0.8 (-5.5; 7.2)
M, age≥75	ITA	6,649	6,653	-4 (-239; 231)	-0.1 (-3.6; 3.5)
F, age≥75	ITA	7,282	7,874	-592 (-852; -331)	-7.5 (-10.8; -4.2)
M, age<75	NET	282	268	14 (-12; 40)	5.2 (-4.5; 14.9)
F, age<75	NET	154	202	-48 (-73; -22)	-23.8 (-36.1; -10.9)
M, age≥75	NET	1,184	1,346	-162 (-261; -62)	-12.0 (-19.4; -4.6)
F, age≥75	NET	1,401	1,775	-374 (-510; -237)	-21.1 (-28.7; -13.4)
M, age<75	POL	4,916	4,391	525 (298; 751)	12.0 (6.8; 17.1)
F, age<75	POL	2,173	1,955	218 (118; 317)	11.2 (6.0; 16.2)
M, age≥75	POL	6,011	5,973	38 (-249; 325)	0.6 (-4.2; 5.4)
F, age≥75	POL	6,813	7,225	-412 (-687; -136)	-5.7 (-9.5; -1.9)
M, age<75	SPA	1,021	1,101	-80 (-139; -20)	-7.3 (-12.6; -1.8)
F, age<75	SPA	489	509	-20 (-56; 16)	-3.9 (-11.0; 3.1)
M, age≥75	SPA	4,129	5,015	-886 (-1083; -688)	-17.7 (-21.6; -13.7)
F, age≥75	SPA	4,023	5,321	-1298 (-1514; -1081)	-24.4 (-28.5; -20.3)
M, age<75	UK	2,272	2,748	-476 (-624; -327)	-17.3 (-22.7; -11.9)
F, age<75	UK	1,530	1,902	-372 (-476; -267)	-19.6 (-25.0; -14.0)
M, age≥75	UK	8,196	11,842	-3646 (-4126; -3165)	-30.8 (-34.8; -26.7)
F, age≥75	UK	9,881	14,676	-4795 (-5308; -4281)	-32.7 (-36.2; -29.2)
M, age<75	ARG	5,614	6,535	-921 (-1331; -510)	-14.1 (-20.4; -7.8)
F, age<75	ARG	3,513	4,286	-773 (-1060; -485)	-18.0 (-24.7; -11.3)
M, age≥75	ARG	7,928	10,996	-3068 (-3692; -2443)	-27.9 (-33.6; -22.2)
F, age≥75	ARG	9,913	15,778	-5865 (-6666; -5063)	-37.2 (-42.2; -32.1)
M, age<75	BRA	16,379	19,617	-3238 (-5093; -1382)	-16.5 (-26.0; -7.0)
F, age<75	BRA	10,839	13,236	-2397 (-3800; -993)	-18.1 (-28.7; -7.5)
M, age≥75	BRA	18,558	24,559	-6001 (-8221; -3780)	-24.4 (-33.5; -15.4)
F, age≥75	BRA	22,418	33,202	-10,784 (-13,727; -7840)	-32.5 (-41.3; -23.6)
M, age<75	CHI	539	706	-167 (-240; -93)	-23.7 (-34.0; -13.2)
F, age<75	CHI	276	331	-55 (-96; -13)	-16.6 (-29.0; -3.9)
M, age≥75	CHI	1,101	1,657	-556 (-701; -410)	-33.6 (-42.3; -24.7)
F, age≥75	CHI	1,293	2,013	-720 (-889; -550)	-35.8 (-44.2; -27.3)
M, age<75	CUB	1,207	1,397	-190 (-285; -94)	-13.6 (-20.4; -6.7)
F, age<75	CUB	769	964	-195 (-264; -125)	-20.2 (-27.4; -13.0)
M, age≥75	CUB	2,378	3,387	-1009 (-1203; -814)	-29.8 (-35.5; -24.0)
F, age≥75	CUB	2,292	3,332	-1040 (-1227; -852)	-31.2 (-36.8; -25.6)
M, age<75	GUA	1,524	2,036	-512 (-1349; 325)	-25.1 (-66.3; 16.0)
F, age<75	GUA	945	1,730	-785 (-1521; -48)	-45.4 (-87.9; -2.8)
M, age≥75	GUA	880	1,443	-563 (-1175; 49)	-39.0 (-81.4; 3.4)
F, age≥75	GUA	719	1,432	-713 (-1320; -105)	-49.8 (-92.2; -7.3)
M, age<75	MEX	25,029	9,177	15,852 (14,409; 17,294)	172.7 (157.0; 188.4)
F, age<75	MEX	13,962	6,144	7818 (6661; 8974)	127.2 (108.4; 146.1)
M, age≥75	MEX	10,159	7,955	2204 (927; 3480)	27.7 (11.7; 43.7)
F, age≥75	MEX	8,127	7,930	197 (-1202; 1596)	2.5 (-15.2; 20.1)
M, age<75	USA	12,264	9,544	2720 (2151; 3288)	28.5 (22.5; 34.5)
F, age<75	USA	9,227	7,512	1715 (1274; 2155)	22.8 (17.0; 28.7)
M, age≥75	USA	15,480	17,048	-1568 (-2490; -645)	-9.2 (-14.6; -3.8)
F, age≥75	USA	16,571	20,220	-3649 (-4644; -2653)	-18.0 (-23.0; -13.1)
M, age<75	JAP	6,318	6,804	-486 (-741; -230)	-7.1 (-10.9; -3.4)
F, age<75	JAP	1,829	2,187	-358 (-468; -247)	-16.4 (-21.4; -11.3)
M, age≥75	JAP	39,097	45,574	-6477 (-7668; -5285)	-14.2 (-16.8; -11.6)

Sex/Age group	Country	Observed deaths	Expected deaths	Absolute difference (95% CI)	Percent difference (95% CI)
F, age≥75	JAP	32,157	40,102	-7945 (-8968; -6921)	-19.8 (-22.4; -17.3)
M, age<75	KOR	2,783	3,408	-625 (-749; -500)	-18.3 (-22.0; -14.7)
F, age<75	KOR	889	1,149	-260 (-318; -201)	-22.6 (-27.7; -17.5)
M, age≥75	KOR	9,429	11,729	-2300 (-2628; -1971)	-19.6 (-22.4; -16.8)
F, age≥75	KOR	9,408	12,395	-2987 (-3335; -2638)	-24.1 (-26.9; -21.3)
M, age<75	AUS	175	364	-189 (-221; -156)	-51.9 (-60.7; -42.9)
F, age<75	AUS	121	267	-146 (-170; -121)	-54.7 (-63.7; -45.3)
M, age≥75	AUS	918	1,529	-611 (-711; -510)	-40.0 (-46.5; -33.4)
F, age≥75	AUS	1,073	2,088	-1015 (-1130; -899)	-48.6 (-54.1; -43.1)

Table 15. Absolute and percent differences in the number of deaths from dementia and Alzheimer disease registered in 2020 relative to the expected deaths, by age group (<75 vs ≥75 years) in countries with population ≥10 million.

Sex/Age group	Country	Observed deaths	Expected deaths	Absolute difference (95% CI)	Percent difference (95% CI)
M, age<75	CZE	282	292	-10 (-47; 27)	-3.4 (-16.1; 9.2)
F, age<75	CZE	269	287	-18 (-49; 13)	-6.3 (-17.1; 4.5)
M, age≥75	CZE	1,236	1,224	12 (-102; 126)	1.0 (-8.3; 10.3)
F, age≥75	CZE	2,566	2,478	88 (-67; 243)	3.6 (-2.7; 9.8)
M, age<75	GER	1,466	1,829	-363 (-520; -205)	-19.8 (-28.4; -11.2)
F, age<75	GER	1,443	1,714	-271 (-414; -127)	-15.8 (-24.2; -7.4)
M, age≥75	GER	18,957	21,914	-2957 (-3961; -1952)	-13.5 (-18.1; -8.9)
F, age≥75	GER	38,827	45,099	-6272 (-7724; -4819)	-13.9 (-17.1; -10.7)
M, age<75	ITA	920	965	-45 (-124; 34)	-4.7 (-12.8; 3.5)
F, age<75	ITA	894	996	-102 (-183; -20)	-10.2 (-18.4; -2.0)
M, age≥75	ITA	10,863	11,230	-367 (-873; 139)	-3.3 (-7.8; 1.2)
F, age≥75	ITA	24,718	24,827	-109 (-921; 703)	-0.4 (-3.7; 2.8)
M, age<75	NET	401	501	-100 (-147; -52)	-20.0 (-29.3; -10.4)
F, age<75	NET	467	511	-44 (-100; 12)	-8.6 (-19.6; 2.3)
M, age≥75	NET	4,435	5,692	-1257 (-1541; -972)	-22.1 (-27.1; -17.1)
F, age≥75	NET	8,976	10,826	-1850 (-2335; -1364)	-17.1 (-21.6; -12.6)
M, age<75	POL	224	214	10 (-18; 38)	4.7 (-8.4; 17.8)
F, age<75	POL	308	334	-26 (-66; 14)	-7.8 (-19.8; 4.2)
M, age≥75	POL	760	684	76 (4; 147)	11.1 (0.6; 21.5)
F, age≥75	POL	2,237	1,804	433 (285; 580)	24.0 (15.8; 32.2)
M, age<75	SPA	862	934	-72 (-153; 9)	-7.7 (-16.4; 1.0)
F, age<75	SPA	865	829	36 (-36; 108)	4.3 (-4.3; 13.0)
M, age≥75	SPA	10,275	11,458	-1183 (-1715; -650)	-10.3 (-15.0; -5.7)
F, age≥75	SPA	24,391	25,725	-1334 (-2153; -514)	-5.2 (-8.4; -2.0)
M, age<75	UK	1,858	2,182	-324 (-533; -114)	-14.8 (-24.4; -5.2)
F, age<75	UK	2,100	2,297	-197 (-433; 39)	-8.6 (-18.9; 1.7)
M, age≥75	UK	25,106	29,338	-4232 (-5642; -2821)	-14.4 (-19.2; -9.6)
F, age≥75	UK	49,322	55,962	-6640 (-8862; -4417)	-11.9 (-15.8; -7.9)
M, age<75	ARG	124	159	-35 (-57; -12)	-22.0 (-35.8; -7.5)
F, age<75	ARG	154	186	-32 (-52; -11)	-17.2 (-28.0; -5.9)
M, age≥75	ARG	783	880	-97 (-180; -13)	-11.0 (-20.5; -1.5)
F, age≥75	ARG	2,030	2,251	-221 (-355; -86)	-9.8 (-15.8; -3.8)
M, age<75	BRA	1,373	1,420	-47 (-235; 141)	-3.3 (-16.5; 9.9)
F, age<75	BRA	1,469	1,461	8 (-149; 165)	0.5 (-10.2; 11.3)
M, age≥75	BRA	8,219	8,465	-246 (-993; 501)	-2.9 (-11.7; 5.9)
F, age≥75	BRA	15,676	16,454	-778 (-1740; 184)	-4.7 (-10.6; 1.1)
M, age<75	CHI	157	165	-8 (-30; 14)	-4.8 (-18.2; 8.5)
F, age<75	CHI	162	155	7 (-12; 26)	4.5 (-7.7; 16.8)
M, age≥75	CHI	1,315	1,141	174 (71; 276)	15.2 (6.2; 24.2)
F, age≥75	CHI	2,719	2,276	443 (299; 586)	19.5 (13.1; 25.7)
M, age<75	CUB	323	314	9 (-24; 42)	2.9 (-7.6; 13.4)
F, age<75	CUB	306	317	-11 (-46; 24)	-3.5 (-14.5; 7.6)
M, age≥75	CUB	1,978	2,253	-275 (-427; -122)	-12.2 (-19.0; -5.4)
F, age≥75	CUB	2,829	3,038	-209 (-404; -13)	-6.9 (-13.3; -0.4)
M, age<75	GUA	9[a]	.	.	.
F, age<75	GUA	17	18	-1 (-7; 5)	-5.6 (-38.9; 27.8)
M, age≥75	GUA	69	71	-2 (-23; 19)	-2.8 (-32.4; 26.8)
F, age≥75	GUA	112	126	-14 (-40; 12)	-11.1 (-31.7; 9.5)
M, age<75	MEX	267	262	5 (-44; 54)	1.9 (-16.8; 20.6)
F, age<75	MEX	303	316	-13 (-62; 36)	-4.1 (-19.6; 11.4)
M, age≥75	MEX	1,156	1,111	45 (-107; 197)	4.1 (-9.6; 17.7)
F, age≥75	MEX	1,967	1,902	65 (-129; 259)	3.4 (-6.8; 13.6)
M, age<75	USA	9,126	8,300	826 (-39; 1691)	10.0 (-0.5; 20.4)
F, age<75	USA	11,459	9,879	1580 (653; 2506)	16.0 (6.6; 25.4)
M, age≥75	USA	73,615	71,022	2593 (-1841; 7027)	3.7 (-2.6; 9.9)
F, age≥75	USA	165,313	155,279	10,034 (3145; 16,922)	6.5 (2.0; 10.9)
M, age<75	JAP	1,148	1,342	-194 (-341; -46)	-14.5 (-25.4; -3.4)
F, age<75	JAP	614	747	-133 (-203; -62)	-17.8 (-27.2; -8.3)
M, age≥75	JAP	13,749	16,870	-3121 (-4042; -2199)	-18.5 (-24.0; -13.0)

Sex/Age group	Country	Observed deaths	Expected deaths	Absolute difference (95% CI)	Percent difference (95% CI)
F, age≥75	JAP	26,167	31,430	-5263 (-6163; -4362)	-16.7 (-19.6; -13.9)
M, age<75	KOR	432	426	6 (-55; 67)	1.4 (-12.9; 15.7)
F, age<75	KOR	216	262	-46 (-75; -16)	-17.6 (-28.6; -6.1)
M, age≥75	KOR	2,896	2,819	77 (-195; 349)	2.7 (-6.9; 12.4)
F, age≥75	KOR	7,096	7,342	-246 (-586; 94)	-3.4 (-8.0; 1.3)
M, age<75	AUS	371	468	-97 (-141; -52)	-20.7 (-30.1; -11.1)
F, age<75	AUS	416	460	-44 (-91; 3)	-9.6 (-19.8; 0.7)
M, age≥75	AUS	4,879	5,354	-475 (-748; -201)	-8.9 (-14.0; -3.8)
F, age≥75	AUS	8,909	9,579	-670 (-1073; -266)	-7.0 (-11.2; -2.8)

[a] Expected deaths were not estimated when observed deaths were <10

Table 16. Absolute and percent differences in the number of deaths from transport accidents registered in 2020 relative to the expected deaths, by age group (<75 vs ≥75 years) in countries with population ≥10 million.

Sex/Age group	Country	Observed deaths	Expected deaths	Absolute difference (95% CI)	Percent difference (95% CI)
M, age<75	CZE	445	466	-21 (-100; 58)	-4.5 (-21.5; 12.4)
F, age<75	CZE	126	129	-3 (-24; 18)	-2.3 (-18.6; 14.0)
M, age≥75	CZE	61	51	10 (-5; 25)	19.6 (-9.8; 49.0)
F, age≥75	CZE	34	36	-2 (-9; 5)	-5.6 (-25.0; 13.9)
M, age<75	GER	1,881	2,003	-122 (-438; 194)	-6.1 (-21.9; 9.7)
F, age<75	GER	497	553	-56 (-128; 16)	-10.1 (-23.1; 2.9)
M, age≥75	GER	492	490	2 (-102; 106)	0.4 (-20.8; 21.6)
F, age≥75	GER	248	266	-18 (-57; 21)	-6.8 (-21.4; 7.9)
M, age<75	ITA	1,658	2,060	-402 (-712; -91)	-19.5 (-34.6; -4.4)
F, age<75	ITA	351	492	-141 (-209; -72)	-28.7 (-42.5; -14.6)
M, age≥75	ITA	445	553	-108 (-216; 0)	-19.5 (-39.1; 0.0)
F, age≥75	ITA	164	215	-51 (-85; -16)	-23.7 (-39.5; -7.4)
M, age<75	NET	353	342	11 (-40; 62)	3.2 (-11.7; 18.1)
F, age<75	NET	110	120	-10 (-27; 7)	-8.3 (-22.5; 5.8)
M, age≥75	NET	138	160	-22 (-50; 6)	-13.8 (-31.2; 3.8)
F, age≥75	NET	68	72	-4 (-15; 7)	-5.6 (-20.8; 9.7)
M, age<75	POL	2,651	2,332	319 (-50; 688)	13.7 (-2.1; 29.5)
F, age<75	POL	563	627	-64 (-140; 12)	-10.2 (-22.3; 1.9)
M, age≥75	POL	207	192	15 (-42; 72)	7.8 (-21.9; 37.5)
F, age≥75	POL	222	171	51 (24; 77)	29.8 (14.0; 45.0)
M, age<75	SPA	1,188	1,344	-156 (-347; 35)	-11.6 (-25.8; 2.6)
F, age<75	SPA	241	318	-77 (-117; -36)	-24.2 (-36.8; -11.3)
M, age≥75	SPA	214	270	-56 (-110; -1)	-20.7 (-40.7; -0.4)
F, age≥75	SPA	83	124	-41 (-59; -22)	-33.1 (-47.6; -17.7)
M, age<75	UK	926	1,147	-221 (-423; -18)	-19.3 (-36.9; -1.6)
F, age<75	UK	223	338	-115 (-163; -66)	-34.0 (-48.2; -19.5)
M, age≥75	UK	165	194	-29 (-80; 22)	-14.9 (-41.2; 11.3)
F, age≥75	UK	109	140	-31 (-54; -7)	-22.1 (-38.6; -5.0)
M, age<75	ARG	1,845	2,874	-1029 (-1623; -434)	-35.8 (-56.5; -15.1)
F, age<75	ARG	387	761	-374 (-474; -273)	-49.1 (-62.3; -35.9)
M, age≥75	ARG	89	133	-44 (-113; 25)	-33.1 (-85.0; 18.8)
F, age≥75	ARG	28	71	-43 (-59; -26)	-60.6 (-83.1; -36.6)
M, age<75	BRA	27,277	25,117	2160 (-2609; 6929)	8.6 (-10.4; 27.6)
F, age<75	BRA	4,994	4,937	57 (-520; 634)	1.2 (-10.5; 12.8)
M, age≥75	BRA	1,033	1,217	-184 (-775; 407)	-15.1 (-63.7; 33.4)
F, age≥75	BRA	353	532	-179 (-286; -71)	-33.6 (-53.8; -13.3)
M, age<75	CHI	1,330	1,360	-30 (-243; 183)	-2.2 (-17.9; 13.5)
F, age<75	CHI	317	346	-29 (-65; 7)	-8.4 (-18.8; 2.0)
M, age≥75	CHI	69	112	-43 (-77; -8)	-38.4 (-68.8; -7.1)
F, age≥75	CHI	33	53	-20 (-28; -11)	-37.7 (-52.8; -20.8)
M, age<75	CUB	495	683	-188 (-288; -87)	-27.5 (-42.2; -12.7)
F, age<75	CUB	108	170	-62 (-86; -37)	-36.5 (-50.6; -21.8)
M, age≥75	CUB	56	97	-41 (-64; -17)	-42.3 (-66.0; -17.5)
F, age≥75	CUB	17	32	-15 (-22; -7)	-46.9 (-68.8; -21.9)
M, age<75	GUA	1,096	1,526	-430 (-779; -80)	-28.2 (-51.0; -5.2)
F, age<75	GUA	202	293	-91 (-132; -49)	-31.1 (-45.1; -16.7)
M, age≥75	GUA	33	67	-34 (-80; 12)	-50.7 (-119.4; 17.9)
F, age≥75	GUA	14	21	-7 (-13; 0)	-33.3 (-61.9; 0.0)
M, age<75	MEX	10,670	11,435	-765 (-2953; 1423)	-6.7 (-25.8; 12.4)
F, age<75	MEX	2,257	2,664	-407 (-672; -141)	-15.3 (-25.2; -5.3)
M, age≥75	MEX	481	620	-139 (-423; 145)	-22.4 (-68.2; 23.4)
F, age≥75	MEX	153	265	-112 (-158; -65)	-42.3 (-59.6; -24.5)
M, age<75	USA	30,270	28,383	1887 (-2612; 6386)	6.6 (-9.2; 22.5)
F, age<75	USA	10,826	10,488	338 (-963; 1639)	3.2 (-9.2; 15.6)
M, age≥75	USA	2,648	2,991	-343 (-1205; 519)	-11.5 (-40.3; 17.4)
F, age≥75	USA	1,415	1,863	-448 (-792; -103)	-24.0 (-42.5; -5.5)
M, age<75	JAP	1,782	1,963	-181 (-390; 28)	-9.2 (-19.9; 1.4)
F, age<75	JAP	490	607	-117 (-161; -72)	-19.3 (-26.5; -11.9)
M, age≥75	JAP	866	982	-116 (-233; 1)	-11.8 (-23.7; 0.1)

Sex/Age group	Country	Observed deaths	Expected deaths	Absolute difference (95% CI)	Percent difference (95% CI)
F, age≥75	JAP	683	710	-27 (-78; 24)	-3.8 (-11.0; 3.4)
M, age<75	KOR	2,291	2,428	-137 (-369; 95)	-5.6 (-15.2; 3.9)
F, age<75	KOR	592	713	-121 (-175; -66)	-17.0 (-24.5; -9.3)
M, age≥75	KOR	698	689	9 (-76; 94)	1.3 (-11.0; 13.6)
F, age≥75	KOR	406	402	4 (-29; 37)	1.0 (-7.2; 9.2)
M, age<75	AUS	897	937	-40 (-184; 104)	-4.3 (-19.6; 11.1)
F, age<75	AUS	265	268	-3 (-40; 34)	-1.1 (-14.9; 12.7)
M, age≥75	AUS	136	136	0 (-33; 33)	0.0 (-24.3; 24.3)
F, age≥75	AUS	57	70	-13 (-25; 0)	-18.6 (-35.7; 0.0)

Table 17. Absolute and percent differences in the number of deaths from suicides registered in 2020 relative to the expected deaths, by age group (<75 vs ≥75 years) in countries with population ≥10 million.

Sex/Age group	Country	Observed deaths	Expected deaths	Absolute difference (95% CI)	Percent difference (95% CI)
M, age<75	CZE	839	829	10 (-119; 139)	1.2 (-14.4; 16.8)
F, age<75	CZE	180	212	-32 (-64; 0)	-15.1 (-30.2; 0.0)
M, age≥75	CZE	170	122	48 (18; 77)	39.3 (14.8; 63.1)
F, age≥75	CZE	35	43	-8 (-17; 1)	-18.6 (-39.5; 2.3)
M, age<75	GER	5,133	5,168	-35 (-662; 592)	-0.7 (-12.8; 11.5)
F, age<75	GER	1,627	1,644	-17 (-187; 153)	-1.0 (-11.4; 9.3)
M, age≥75	GER	1,819	1,745	74 (-187; 335)	4.2 (-10.7; 19.2)
F, age≥75	GER	642	580	62 (-11; 135)	10.7 (-1.9; 23.3)
M, age<75	ITA	2,160	2,182	-22 (-310; 266)	-1.0 (-14.2; 12.2)
F, age<75	ITA	615	656	-41 (-120; 38)	-6.2 (-18.3; 5.8)
M, age≥75	ITA	682	617	65 (-39; 169)	10.5 (-6.3; 27.4)
F, age≥75	ITA	161	161	0 (-26; 26)	0.0 (-16.1; 16.1)
M, age<75	NET	1,091	1,136	-45 (-218; 128)	-4.0 (-19.2; 11.3)
F, age<75	NET	518	570	-52 (-132; 28)	-9.1 (-23.2; 4.9)
M, age≥75	NET	137	135	2 (-34; 38)	1.5 (-25.2; 28.1)
F, age≥75	NET	78	71	7 (-9; 23)	9.9 (-12.7; 32.4)
M, age<75	POL	3,685	3,329	356 (-163; 875)	10.7 (-4.9; 26.3)
F, age<75	POL	542	485	57 (-7; 121)	11.8 (-1.4; 24.9)
M, age≥75	POL	273	215	58 (-10; 126)	27.0 (-4.7; 58.6)
F, age≥75	POL	59	63	-4 (-17; 9)	-6.3 (-27.0; 14.3)
M, age<75	SPA	2,341	2,180	161 (-134; 456)	7.4 (-6.1; 20.9)
F, age<75	SPA	834	807	27 (-76; 130)	3.3 (-9.4; 16.1)
M, age≥75	SPA	590	569	21 (-80; 122)	3.7 (-14.1; 21.4)
F, age≥75	SPA	182	172	10 (-21; 41)	5.8 (-12.2; 23.8)
M, age<75	UK	3,918	3,860	58 (-618; 734)	1.5 (-16.0; 19.0)
F, age<75	UK	1,235	1,214	21 (-172; 214)	1.7 (-14.2; 17.6)
M, age≥75	UK	270	311	-41 (-152; 70)	-13.2 (-48.9; 22.5)
F, age≥75	UK	104	114	-10 (-44; 24)	-8.8 (-38.6; 21.1)
M, age<75	ARG	2,123	2,445	-322 (-864; 220)	-13.2 (-35.3; 9.0)
F, age<75	ARG	477	569	-92 (-215; 31)	-16.2 (-37.8; 5.4)
M, age≥75	ARG	183	219	-36 (-130; 58)	-16.4 (-59.4; 26.5)
F, age≥75	ARG	20	28	-8 (-23; 7)	-28.6 (-82.1; 25.0)
M, age<75	BRA	10,282	10,229	53 (-1831; 1937)	0.5 (-17.9; 18.9)
F, age<75	BRA	2,847	2,828	19 (-428; 466)	0.7 (-15.1; 16.5)
M, age≥75	BRA	569	594	-25 (-297; 247)	-4.2 (-50.0; 41.6)
F, age≥75	BRA	118	117	1 (-52; 54)	0.9 (-44.4; 46.2)
M, age<75	CHI	1,232	1,400	-168 (-431; 95)	-12.0 (-30.8; 6.8)
F, age<75	CHI	269	313	-44 (-100; 12)	-14.1 (-31.9; 3.8)
M, age≥75	CHI	90	108	-18 (-60; 24)	-16.7 (-55.6; 22.2)
F, age≥75	CHI	11	12	-1 (-7; 5)	-8.3 (-58.3; 41.7)
M, age<75	CUB	965	918	47 (-76; 170)	5.1 (-8.3; 18.5)
F, age<75	CUB	220	235	-15 (-47; 17)	-6.4 (-20.0; 7.2)
M, age≥75	CUB	317	263	54 (7; 100)	20.5 (2.7; 38.0)
F, age≥75	CUB	54	54	0 (-10; 10)	0.0 (-18.5; 18.5)
M, age<75	GUA	396	499	-103 (-233; 27)	-20.6 (-46.7; 5.4)
F, age<75	GUA	167	164	3 (-53; 59)	1.8 (-32.3; 36.0)
M, age≥75	GUA	9[a]	.	.	.
F, age≥75	GUA	2[a]	.	.	.
M, age<75	MEX	6,141	5,609	532 (-702; 1766)	9.5 (-12.5; 31.5)
F, age<75	MEX	1,402	1,307	95 (-189; 379)	7.3 (-14.5; 29.0)
M, age≥75	MEX	183	204	-21 (-166; 124)	-10.3 (-81.4; 60.8)
F, age≥75	MEX	13	13	0 (-28; 28)	0.0 (-215.4; 215.4)
M, age<75	USA	32,650	34,876	-2226 (-7903; 3451)	-6.4 (-22.7; 9.9)
F, age<75	USA	8,906	10,462	-1556 (-3084; -27)	-14.9 (-29.5; -0.3)
M, age≥75	USA	3,900	4,017	-117 (-1275; 1041)	-2.9 (-31.7; 25.9)
F, age≥75	USA	521	604	-83 (-290; 124)	-13.7 (-48.0; 20.5)
M, age<75	JAP	11,407	10,485	922 (-618; 2462)	8.8 (-5.9; 23.5)
F, age<75	JAP	5,299	4,071	1228 (675; 1780)	30.2 (16.6; 43.7)
M, age≥75	JAP	2,151	1,967	184 (-231; 599)	9.4 (-11.7; 30.5)

Sex/Age group	Country	Observed deaths	Expected deaths	Absolute difference (95% CI)	Percent difference (95% CI)
F, age≥75	JAP	1,355	1,262	93 (-120; 306)	7.4 (-9.5; 24.2)
M, age<75	KOR	7,832	7,618	214 (-880; 1308)	2.8 (-11.6; 17.2)
F, age<75	KOR	3,464	2,721	743 (308; 1177)	27.3 (11.3; 43.3)
M, age≥75	KOR	1,261	1,529	-268 (-593; 57)	-17.5 (-38.8; 3.7)
F, age≥75	KOR	639	777	-138 (-299; 23)	-17.8 (-38.5; 3.0)
M, age<75	AUS	2,187	2,456	-269 (-690; 152)	-11.0 (-28.1; 6.2)
F, age<75	AUS	692	803	-111 (-238; 16)	-13.8 (-29.6; 2.0)
M, age≥75	AUS	197	227	-30 (-106; 46)	-13.2 (-46.7; 20.3)
F, age≥75	AUS	63	71	-8 (-29; 13)	-11.3 (-40.8; 18.3)

[a] Expected deaths were not estimated when observed deaths were <10

Table 18. Absolute and percent differences in the number of deaths from ill-defined causes registered in 2020 relative to the expected deaths, by age group (<75 vs ≥75 years) in countries with population ≥10 million.

Sex/Age group	Country	Observed deaths	Expected deaths	Absolute difference (95% CI)	Percent difference (95% CI)
M, age<75	CZE	990	739	251 (180; 321)	34.0 (24.4; 43.4)
F, age<75	CZE	456	330	126 (98; 153)	38.2 (29.7; 46.4)
M, age≥75	CZE	852	338	514 (476; 551)	152.1 (140.8; 163.0)
F, age≥75	CZE	1,378	584	794 (751; 836)	136.0 (128.6; 143.2)
M, age<75	GER	12,559	13,621	-1062 (-1887; -236)	-7.8 (-13.9; -1.7)
F, age<75	GER	5,262	5,047	215 (-58; 488)	4.3 (-1.1; 9.7)
M, age≥75	GER	7,806	8,542	-736 (-1299; -172)	-8.6 (-15.2; -2.0)
F, age≥75	GER	9,518	10,283	-765 (-1250; -279)	-7.4 (-12.2; -2.7)
M, age<75	ITA	3,960	1,872	2088 (1933; 2242)	111.5 (103.3; 119.8)
F, age<75	ITA	1,572	762	810 (766; 853)	106.3 (100.5; 111.9)
M, age≥75	ITA	6,676	3,965	2711 (2427; 2994)	68.4 (61.2; 75.5)
F, age≥75	ITA	13,724	9,166	4558 (4278; 4837)	49.7 (46.7; 52.8)
M, age<75	NET	2,003	1,637	366 (258; 473)	22.4 (15.8; 28.9)
F, age<75	NET	988	811	177 (127; 226)	21.8 (15.7; 27.9)
M, age≥75	NET	2,413	1,849	564 (445; 682)	30.5 (24.1; 36.9)
F, age≥75	NET	3,590	3,025	565 (423; 706)	18.7 (14.0; 23.3)
M, age<75	POL	15,442	15,781	-339 (-1884; 1206)	-2.1 (-11.9; 7.6)
F, age<75	POL	5,104	4,656	448 (83; 812)	9.6 (1.8; 17.4)
M, age≥75	POL	9,751	8,789	962 (2; 1921)	10.9 (0.0; 21.9)
F, age≥75	POL	19,398	18,298	1100 (50; 2149)	6.0 (0.3; 11.7)
M, age<75	SPA	1,492	1,983	-491 (-664; -317)	-24.8 (-33.5; -16.0)
F, age<75	SPA	573	757	-184 (-233; -134)	-24.3 (-30.8; -17.7)
M, age≥75	SPA	2,686	2,341	345 (146; 543)	14.7 (6.2; 23.2)
F, age≥75	SPA	4,669	4,061	608 (419; 796)	15.0 (10.3; 19.6)
M, age<75	UK	905	1,107	-202 (-304; -99)	-18.2 (-27.5; -8.9)
F, age<75	UK	499	480	19 (-30; 68)	4.0 (-6.2; 14.2)
M, age≥75	UK	4,407	3,540	867 (609; 1124)	24.5 (17.2; 31.8)
F, age≥75	UK	12,131	10,110	2021 (1579; 2462)	20.0 (15.6; 24.4)
M, age<75	ARG	6,249	5,321	928 (549; 1306)	17.4 (10.3; 24.5)
F, age<75	ARG	3,315	2,797	518 (328; 707)	18.5 (11.7; 25.3)
M, age≥75	ARG	5,561	4,825	736 (387; 1084)	15.3 (8.0; 22.5)
F, age≥75	ARG	8,190	6,995	1195 (788; 1601)	17.1 (11.3; 22.9)
M, age<75	BRA	34,870	25,631	9239 (6924; 11553)	36.0 (27.0; 45.1)
F, age<75	BRA	16,292	11,800	4492 (3687; 5296)	38.1 (31.2; 44.9)
M, age≥75	BRA	17,561	15,147	2414 (917; 3910)	15.9 (6.1; 25.8)
F, age≥75	BRA	20,906	18,100	2806 (1660; 3951)	15.5 (9.2; 21.8)
M, age<75	CHI	777	666	111 (36; 185)	16.7 (5.4; 27.8)
F, age<75	CHI	367	310	57 (19; 94)	18.4 (6.1; 30.3)
M, age≥75	CHI	691	682	9 (-66; 84)	1.3 (-9.7; 12.3)
F, age≥75	CHI	1,085	1,044	41 (-59; 141)	3.9 (-5.7; 13.5)
M, age<75	CUB	434	441	-7 (-54; 40)	-1.6 (-12.2; 9.1)
F, age<75	CUB	183	170	13 (-7; 33)	7.6 (-4.1; 19.4)
M, age≥75	CUB	230	226	4 (-23; 31)	1.8 (-10.2; 13.7)
F, age≥75	CUB	244	245	-1 (-28; 26)	-0.4 (-11.4; 10.6)
M, age<75	GUA	3,763	3,572	191 (-322; 704)	5.3 (-9.0; 19.7)
F, age<75	GUA	2,767	2,890	-123 (-523; 277)	-4.3 (-18.1; 9.6)
M, age≥75	GUA	3,402	2,968	434 (-2; 870)	14.6 (-0.1; 29.3)
F, age≥75	GUA	3,267	3,307	-40 (-478; 398)	-1.2 (-14.5; 12.0)
M, age<75	MEX	1,564	1,301	263 (136; 389)	20.2 (10.5; 29.9)
F, age<75	MEX	933	857	76 (-14; 166)	8.9 (-1.6; 19.4)
M, age≥75	MEX	3,818	2,807	1011 (772; 1249)	36.0 (27.5; 44.5)
F, age≥75	MEX	3,874	3,219	655 (393; 916)	20.3 (12.2; 28.5)
M, age<75	USA	14,401	9,293	5108 (3690; 6525)	55.0 (39.7; 70.2)
F, age<75	USA	8,920	4,943	3977 (3198; 4755)	80.5 (64.7; 96.2)
M, age≥75	USA	9,983	8,455	1528 (220; 2835)	18.1 (2.6; 33.5)
F, age≥75	USA	15,407	12,132	3275 (1653; 4896)	27.0 (13.6; 40.4)
M, age<75	JAP	10,391	9,908	483 (4; 961)	4.9 (0.0; 9.7)
F, age<75	JAP	3,011	2,979	32 (-153; 217)	1.1 (-5.1; 7.3)
M, age≥75	JAP	42,536	41,997	539 (-952; 2030)	1.3 (-2.3; 4.8)

Sex/Age group	Country	Observed deaths	Expected deaths	Absolute difference (95% CI)	Percent difference (95% CI)
F, age≥75	JAP	103,867	106,663	-2796 (-5084; -507)	-2.6 (-4.8; -0.5)
M, age<75	KOR	5,983	4,454	1529 (1232; 1825)	34.3 (27.7; 41.0)
F, age<75	KOR	1,793	1,298	495 (407; 582)	38.1 (31.4; 44.8)
M, age≥75	KOR	9,186	7,637	1549 (1085; 2012)	20.3 (14.2; 26.3)
F, age≥75	KOR	16,921	14,400	2521 (1949; 3092)	17.5 (13.5; 21.5)
M, age<75	AUS	963	412	551 (484; 617)	133.7 (117.5; 149.8)
F, age<75	AUS	458	271	187 (142; 231)	69.0 (52.4; 85.2)
M, age≥75	AUS	594	377	217 (155; 278)	57.6 (41.1; 73.7)
F, age≥75	AUS	973	706	267 (173; 360)	37.8 (24.5; 51.0)

Figure 2. Sensitivity analysis: comparison of the relative difference between observed and expected deaths (P-score) in 2019 vs 2020.

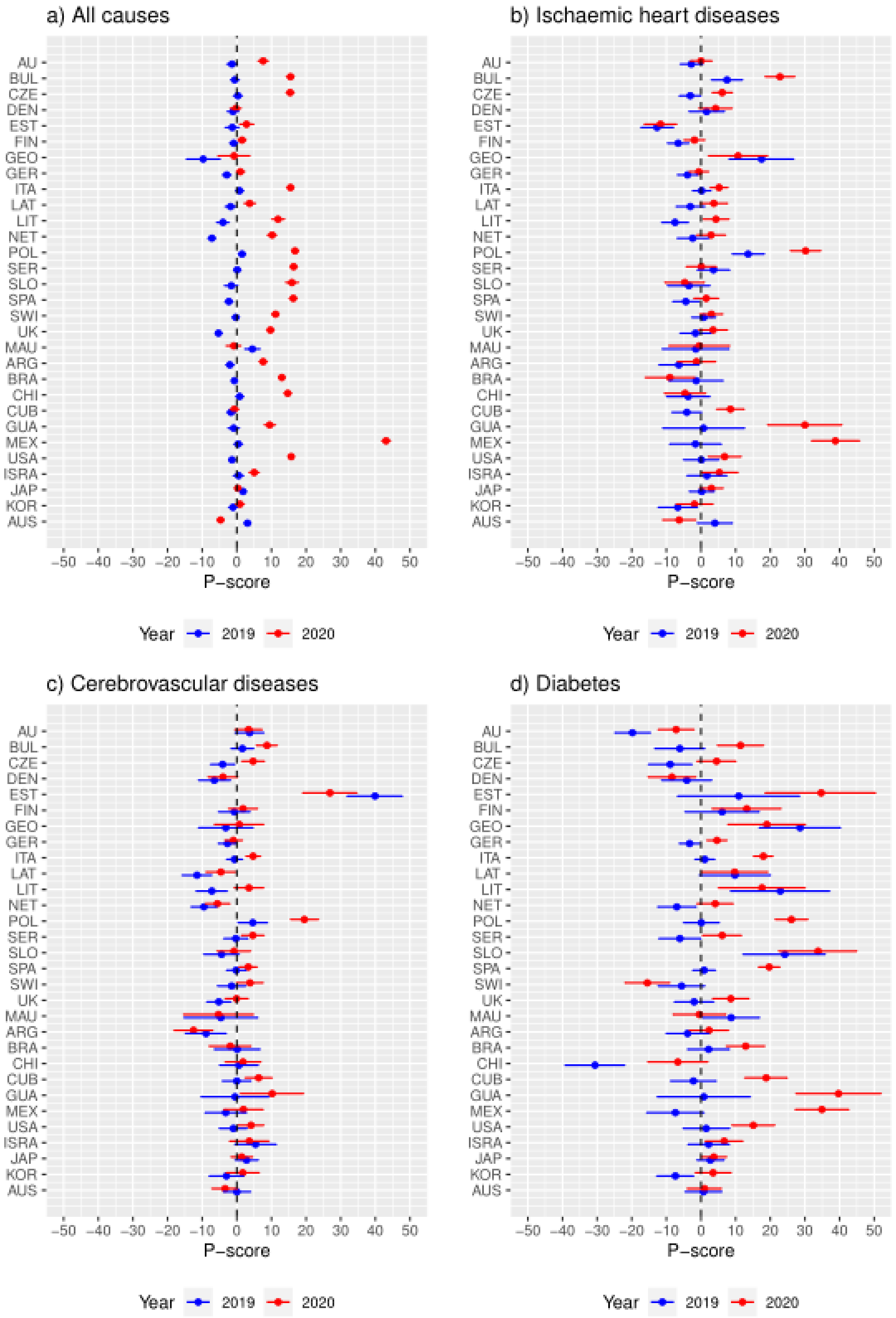

Supplement: Supplementary file 1 — Supplementary file1 (PDF 1150 KB) [file 10654_2023_1044_MOESM1_ESM.pdf]
